# Systematics of the Neotropical genus *Catharylla* Zeller (Lepidoptera, Pyralidae s. l., Crambinae)

**DOI:** 10.3897/zookeys.375.6222

**Published:** 2014-01-30

**Authors:** Théo Léger, Bernard Landry, Matthias Nuss, Richard Mally

**Affiliations:** 1 Muséum d’histoire naturelle de Genève, Route de Malagnou, 1, CH-1211, Geneva, Switzerland; 2 Senckenberg Natural History Collections, Museum of Zoology, Königsbrücker Landstraße 159, 01109 Dresden, Germany

**Keywords:** *Argyria*, Argyriini, Atlantic forest, biogeography, Crambini, *Micrelephas*, morphology, new species, phylogeny, Pyraloidea, taxonomy

## Abstract

The Neotropical genus *Catharylla* Zeller, 1863 (type species: *Crambus tenellus* Zeller, 1839) is redescribed. *Catharylla contiguella* Zeller, 1872, *C. interrupta* Zeller, 1866 and *Myelois sericina* Zeller, 1881, included by Munroe (1995) in *Catharylla*, are moved to *Argyria* Hübner. *Catharylla paulella* Schaus, 1922 and *C. tenellus* (Zeller, 1839) are redescribed. Six new species are described by Léger and Landry: *C. bijuga*, *C. chelicerata*, *C. coronata*, *C. gigantea*, *C. mayrabonillae* and *C. serrabonita*. The phylogenetic relationships were investigated using morphological as well as molecular data (COI, wingless, EF-1α genes). The median and subterminal transverse lines of the forewing as well as the short anterior and posterior apophyses of the female genitalia are characteristic of the genus. The monophyly of *Catharylla* was recovered in all phylogenetic analyses of the molecular and the combined datasets, with three morphological apomorphies highlighted. Phylogenetic analyses of the morphology of the two sexes recovered three separate species groups within *Catharylla*: the *chelicerata*, the *mayrabonillae*, and the *tenellus* species groups. The possible position of *Micrelephas* Schaus, 1922 as sister to *Catharylla*, based on both morphological and molecular data, and the status of tribe Argyriini are discussed. The biogeographical data indicate that the *chelicerata* species group is restricted to the Guyanas and the Amazonian regions whereas the *tenellus* group is restricted to the Atlantic Forest in the South-Eastern part of Brazil. The *mayrabonillae* group is widespread from Costa Rica to South Bolivia with an allopatric distribution of the two species. COI barcode sequences indicate relatively strong divergence within *C. bijuga*, *C. mayrabonillae*, *C. serrabonita* and *C. tenellus*.

## Introduction

Pyraloidea is one of the largest superfamilies of the order Lepidoptera. The monophyly of the group and that of its two main lineages, the Pyraliformes and Crambiformes (or Pyralidae and Crambidae, depending on authors), are well supported by morphological characters (Börner 1925, Hasenfuß 1960, Minet 1982) and molecular investigations (Regier et al. 2009, 2012; Mutanen et al. 2010). The abdominal tympanal organs represent a distinctive autapomorphy for the superfamily (Munroe and Solis 1998). The phallus attached medially to the juxta, the crambine-type tympanal organs, as well as the presence of a hair tuft on the dorsal hindwing cubital stem support the monophyly of subfamily Crambinae (Landry 1995). This is corroborated by Regier et al. (2012) based on sequences of several genes, but only three taxa and two of them Crambini. The only available, although partial, phylogenetic analysis of the Crambinae Latreille, 1810 is that of Landry (1995), which, however, did not include *Catharylla*. The relationships of *Catharylla* with other genera of Crambinae are not known, except for the placement of the genus in the Argyriini by Munroe (1995) together with *Argyria* Hübner, 1818, *Vaxi* Bleszynski, 1962 and *Urola* Walker, 1863. This placement was based on a suggestion by Bleszynski (1962: 12) that *Catharylla* may be closely related to *Vaxi* (Munroe 1995: 161). Tribe Argyriini was thought to be monophyletic on the basis of the snow white color of the wings, the broad forewings, and the short labial palpi (Landry 1995, Munroe 1995), but its phylogenetic relationships remain unresolved. Stanislas Bleszynski investigated *Catharylla*, but he never published his findings, probably due to his accidental death in 1969.

*Catharylla* moths are of medium size, with snow white to cream-colored wings with two ochreous to brown transverse lines on the forewings. Species of *Catharylla* occur in tropical Central and South America. Nothing is known on the immature stages and biology. The original description of Zeller (1863) gives little information and was restricted to external features. Munroe (1995) included five species in *Catharylla* (*Catharylla contiguella*, *Catharylla interrupta*, *Catharylla paulella*, *Catharylla sericina*, *Catharylla tenellus*), but these share no evident common characteristics other than the snow white color of the wings.

In this work, *Catharylla* is revised using both morphological and molecular data. Phylogenetic relationships within *Catharylla* and with putatively related taxa as well as the distribution of each species along with biogeographical hypotheses are analysed.

## Material and methods

### Morphological investigations
Material

*Catharylla* and outgroup taxa investigated were borrowed from museums and private collectors as listed in Table 1, which also gives the acronyms used throughout the text.

**Table 1.** Collections from which *Catharylla* specimens were borrowed.

Acronym	Museum or Collection	Locality	Number of specimens
**AMNH**	American Museum of Natural History	USA, New York, New York	1
**Becker Coll.**	Collection of V. O. Becker	Brazil, Bahia, Camacan	57
**BMNH**	Natural History Museum	Great Britain, London	34
**CMNH**	Carnegie Museum of Natural History	USA, Pennsylvania, Pittsburgh	9
**CNC**	Canadian National Collection of Insects, Arachnids and Nematodes	Canada, Ontario, Ottawa	14
**ISZP**	Institute of Systematic Zoology	Poland, Krakow	9
**INBio**	Instituto Nacional de Biodiversidad	Costa Rica, Santo Domingo de Heredia	5
**MHNG**	Muséum d’histoire naturelle de Genève	Switzerland, Geneva	28
**NMW**	Naturhistorisches Museum Wien	Austria, Vienna	4
**Schouten Coll.**	Collection R. T. A. Schouten	Netherlands, The Hague	6
**SMNS**	Staatliches Museum für Naturkunde, Stuttgart	Germany, Stuttgart	1
**SMTD**	Staatliches Museum für Tierkunde, Dresden	Germany, Dresden	1
**USNM**	National Museum of Natural History	USA, D. C., Washington	33

Several of the MHNG specimens were kindly given to this institution by collaborators mentioned in the acknowledgments section. The specimens were usually well preserved, the color being sometimes faded. Many specimens from the BMNH were dissected by S. Bleszynski. Unfortunately, his preparations were usually poorly made and badly mounted, sometimes hampering the investigation of genitalia characters.

The dissection and slide mounting methods follow Landry (1995). Genitalia pictures were taken with a Leica MZ APO, a JVC digital camera (KY-F70B), and Auto-Montage version 4.02.0014. The adult pictures were made with a NIKON D300 and a 105 mm Micro NIKON f/2.8G AF-S VR lens. The images were enhanced with Adobe Photoshop Elements.

Apart from the eight *Catharylla* species, four additional crambine species were included in the dataset for phylogenetic analyses. The material investigated to build the morphological matrix is reported below in Table 2.

**Table 2.** Material used for the morphology-based phylogenetic analysis of *Catharylla* species and related genera.

Subfamily	Genus Species	Sex	Collecting locality	Collection	Dissection number
Crambinae	*Argyria lacteella*	M	USA, Florida, Lake Placid, Archbold Biological Station	MHNG	BL 064
Crambinae	*Argyria lacteella*	F	USA, Florida, Lake Placid, Archbold Biological Station	MHNG	BL 067
Crambinae	*Argyria lacteella*	F	Brazil, Bahia, Camacan, Serra Bonita Reserve	MHNG	TL 15 (wing prep.)
Crambinae	*Catharylla bijuga*	M	French Guiana, Saint-Jean-du-Maroni	BMNH	BL 1719
Crambinae	*Catharylla bijuga*	F	Suriname, Sipaliwini District, Thibiti area, partly swampy, primary forest on hilly slopes, ca. 2km from river	Schouten Coll.	BL 1732
Crambinae	*Catharylla chelicerata*	M	Brazil, Reserva Ducke, km. 26 Manaus–Itacoatiara Highway	CNC	BL 1721
Crambinae	*Catharylla chelicerata*	F	Brazil, Reserva Ducke, km. 26 Manaus–Itacoatiara Highway	CNC	BL 1711
Crambinae	*Catharylla chelicerata*	M	French Guiana, 36 km SE Roura (Camp Patawa)	MHNG	MHNG 6272 (wing prep.)
Crambinae	*Catharylla coronata*	M	Brazil, Espirito Santo, Linhares, 40 m	Becker Coll.	BL 1743
Crambinae	*Catharylla coronata*	F	Brazil, Rio Negro, 900 m	ISZP	BL 1731
Crambinae	*Catharylla gigantea*	M	Guyana, Potaro	BMNH	BL 1716
Crambinae	*Catharylla gigantea*	F	French Guiana, Saint-Jean-du-Maroni	BMNH	Pyralidae Brit. Mus. Slide N° 11342
Crambinae	*Catharylla mayrabonillae*	M	Peru, Agnaytia, Huallaga, Peru, 400 m	CNC	BL 1724
Crambinae	*Catharylla mayrabonillae*	F	French Guiana, Saint-Jean-du-Maroni	BMNH	BL 1720
Crambinae	*Catharylla paulella*	M	Bolivia, Provincia del Sara, 450 m	BMNH	Pyralidae Brit. Mus. Slide N° 15890
Crambinae	*Catharylla paulella*	F	Brazil, Mato Grosso, Urucum, 15 miles South of Columbá, 650 feet	BMNH	BL 1712
Crambinae	*Catharylla serrabonita*	M	Brazil, Espirito Santo, Linhares, 40 m	USNM	BL 1745
Crambinae	*Catharylla serrabonita*	F	Brazil, Espirito Santo, Linhares, 40 m	Becker Coll.	BL 1759
Crambinae	*Catharylla serrabonita*	M	Brazil, Bahia, Camacan, Serra Bonita Reserve	MHNG	TL 8 (wing prep.)
Crambinae	*Catharylla tenellus*	M	Brazil, Minas Gerais, Caraça, 1300 m	Becker Coll.	BL 1746
Crambinae	*Catharylla tenellus*	F	Brazil, São Paulo, Bertioga, 5 m	Becker Coll.	BL 1742
Crambinae	*Crambus uliginosellus*	M	Germany, Oberstdorf, Allgäu	SMTD	MTD prep. N°327
Crambinae	*Crambus uliginosellus*	F	Switzerland, St. Gallen	SMTD	MTD prep. N°329
Crambinae	*Crambus pascuella*	M	Germany, Coswig, Dresden	SMTD	MTD prep. N°325
Crambinae	*Crambus pascuella*	F	Germany, Koetzschenbroda, Dresden	SMTD	MTD prep. N°326
Crambinae	*Micrelephas pictellus*	M	Brazil, Bahia, Camacan, 400–700 m	MNHG	MHNG ENTO N°2831
Crambinae	*Micrelephas pictellus*	F	Panama, Barro Colorado Island	MNHG	MHNG ENTO N°2829
Crambinae	*Micrelephas pictellus*	M	Brazil, Bahia, Camacan, Serra Bonita Reserve	MNHG	TL 17 (wing prep.)

### Taxonomy

The types of the two species *Catharylla contiguella* Zeller, 1872, and *Catharylla interrupta* Zeller, 1866 could not be found. With the help of the descriptions and illustrations, they were excluded from *Catharylla* because of the forewing pattern, which is like that of *Argyria* Hübner, 1818, with only one median transverse line. For *Catharylla sericina* Zeller, 1881, based on the description and a photograph of the type in the BMNH, the species was rejected from *Catharylla* based on the elongated forewing shape and the silvery white pattern without transverse lines. For *Catharylla paulella* Schaus, 1922, a photograph of the habitus and the genitalia of the female type from the USNM allowed to find other specimens of the same species; the male and female were then associated based on wing pattern. For *Catharylla tenellus* Zeller, 1839, a photograph of the habitus and the genitalia of the female type were available and the male of the species was associated based on wing pattern. For the descriptions, we followed the nomenclature and terminology used by Landry (1995), except for the use of the term phallus (see Kristensen 2003). New species were recognized based on major differences in male and female genitalia.

The following measurements were made with the use of an ocular micrometer: length of labial palpus (base of segment I to apex of segment III), diameter of eye (greatest vertical width), length of forewing (from base to apex), length of uncus (from tegumen-uncus junction to apex of uncus), length of tegumen connection (from tegumen arms connection medially to tegumen-uncus junction), length of papillae anales (dorso-ventral length), lengths of anterior and posterior apophyses (from base to apex).

Regarding the holotype data, the information was copied exactly as found on the labels with vertical slashes to express changes of lines. Abbreviations are spelled out in square brackets. We assume that the labels are rectangular and white, and that the text is in black ink, otherwise differences are indicated in brackets. Paratype data are reported by country in alphabetical order and the information is recorded without indication of line change. Collecting localities are reported as written on labels, with a question-mark when the locality could not be recovered. Dates and collectors’ information were standardized and the latter placed in parentheses. The specimen depositories are reported with the use of the corresponding acronyms.

### Biogeographical investigations

The coordinates of the localities were found using Google Earth (2011). The localities that were not registered in Google Earth were localized more or less precisely with the help of internet search engines or with gazeeters from the GEOnet Names Server (GNS) of the National Geospatial Intelligence Agency (http://earth-info.nga.mil/gns/html/). The localities were reported on a text file (*.txt) and loaded on a map using DIVA-GIS 7.4.0.1 (Hijmans et al. 2011). Distances between localities were calculated with Google Earth. The localities and their coordinates are reported in Appendix I - Table 1. We refer to the provinces of Morrone (2006) in the biogeography section.

### Molecular investigations
Material

The genes investigated are the mitochondrial COI gene (1474 bp) and the two nuclear genes wingless (353 bp) and EF-1α (679 bp). These genes show different rates of substitution through time, with COI >> wingless > EF-1α (Wahlberg and Wheat 2008). COI performs well at the species level and the two nuclear genes recover accurate phylogenies in deeper nodes of Lepidoptera (Wahlberg and Wheat 2008).

Specimens used for molecular investigations are listed in Table 3, with the sequencing success for the different investigated genes. Specimens were preserved in 95% ethanol under cool conditions until molecular investigations, or were pinned and dried. Sequences for the additional species were obtained from GenBank (see Table 3).

**Table 3.** List of the material used in the molecular work with voucher numbers, database of origin, collecting depository, and sequencing results for each gene. LEP references refer to the Lepidoptera DNA database of M. Nuss at the SMTD. BC MTD references refer to the barcode sequence voucher of the Barcoding Of Life Database (BOLD). HG references refer to the European Nucleotide Archive. Amplicon length (in basepairs) is given in brackets. The sequences with an asterisk were those used to build the datasets used in the phylogenetic analyses.

species	Voucher number	Collecting locality and specimen depository	sequencing results			
			COI		wingless	EF-1α
			1st part	2nd part		
*Catharylla*						
*Catharylla bijuga*	-	French Guiana, Roura, road to Crique Gabrielle, 3.6 km East Roura (AMNH)	BC MTD 01839 (1–654)			
*Catharylla bijuga*	-	Brazil, Amazonas, Parque nacional do Jaú, 1°57'S, 61°49'W (USNM)	BC MTD 01840 (1–654)*			
*Catharylla chelicerata*	LEP 963	French Guiana, 36 km SE Roura (Camp Patawa) (MHNG)	BC MTD 01703 (1–654)			
*Catharylla chelicerata*	-	French Guiana, 36 km SE Roura (Camp Patawa) (MHNG)	BC MTD 01704 (1–654)			
*Catharylla chelicerata*	LEP 1290	French Guiana, 600, Parcelles CIRAD de Combi, plantations expérimentales, pk 1.8 5°18''N, 52°55'30''W (MHNG)	HG793015 (1–1474)*		HG793008 (46–353)*	HG793003 (1–452)*
*Catharylla coronata*	-	Brazil, Espiritu Santo, Linhares, 40m (USNM)	BC MTD 01890 (1–654)*			
*Catharylla mayrabonillae*	LEP 1126	Peru, Huánuco, Rio Llullapichis, Panguana, 74,945°W / 9,614°S (SMTD)	HG793014 (6–1474)*		HG793009 (1–353)*	HG793004 (1–199)*
*Catharylla mayrabonillae*	-	Costa Rica, Alajuela, Area de Conservacion Guanacaste, Estacion Caribe (INBio)	07-SRNP-113921 (1–654)			
*Catharylla paulella*	LEP 965	Brazil, Sao Paulo, Sao Luiz do Paraitinga, 900 m, 23°20'S, 45°06'W(V. O. Becker n°132357) (Becker Coll.)	HG793016 (1–978)*			
*Catharylla serrabonita*	LEP 970	Brazil, Bahia, Porto Seguro, A. d'Ajuda, 16°27'S, 39°03'W, 20m (V. O. Becker n°144140) (Becker Coll.)	BC MTD 01887 (1–654)			
*Catharylla serrabonita*	LEP 979	Brazil, Bahia, Camacan, Reserva Serra Bonita, 800m (MHNG)	HG793017 (35–556)*	HG793018 (751–1333)*	HG793010 (734–1474)*	HG793005 (106–679)*
*Catharylla serrabonita*	-	Brazil, Espiritu Santo, Linhares, 40m (USNM)	BC MTD 01843 (1–654)			
*Catharylla tenellus*	-	Brazil, Bahia, Porto Seguro, A. d'Ajuda, 16°27'S, 39°03'W, 20m (V. O. Becker n°142784) (Becker Coll.)	BC MTD 01708 (15–654)			
*Catharylla tenellus*	LEP 973	Brazil, Bahia, Porto Seguro, A. d'Ajuda, 16°27'S, 39°03'W, 20m (V. O. Becker n°144140) (Becker Coll.)	BC MTD 01709 (1–654)		HG793011 (23–353)*	
*Catharylla tenellus*	-	Brazil, Bahia, Porto Seguro, A. d'Ajuda, 16°27'S, 39°03'W, 20m (V. O. Becker n°140808) (Becker Coll.)	BC MTD 01710 (1–654)			
*Catharylla tenellus*	-	Brazil, São Paulo, Ubatuba, Picinguaba, 23°22'S, 44°50'W (Becker Coll.)	BC MTD 01842 (1–654)			
*Catharylla tenellus*	LEP 972	Brazil, Bahia, Porto Seguro, A. d'Ajuda, 16°27'S, 39°03'W, 20m (V. O. Becker n°140808) (Becker Coll.)	BC MTD 01888 (1–654)*	HG793020 (792–1473)*		
Other Crambinae						
*Argyria lacteella*	LEP 976	Brazil, Bahia, Camacan, Reserva Serra Bonita, 800m (MHNG)	HG793013 (6–689; 709–1474)*		HG793006 (57–355)*	HG793001 (1–654)*
*Crambus pascuella*	-	USA: North Carolina, Swain County, 1720m, 35°35'45''N, 83°27'42''W	GU089400 (1–657)*			
*Crambus uliginosellus*	-	unknown	GU828691 (1–668)*	GU828487 (716–1474)*	GU829571 (1–353)*	GU829302 (269–679)*
*Micrelephas pictellus*	LEP 977	Brazil, Bahia, Camacan, Reserva Serra Bonita, 800m (MHNG)	HG793012 (26–1474)*		HG793007 (1–353)*	HG793002 (1–626)*

For *Catharylla* specimens collected no more than twenty years ago, a leg was sent to the Canadian Centre for DNA Barcoding (CCDB) at the Biodiversity Institute of Ontario (BIO) in Guelph. The barcode sequences of *Catharylla* are reported in each species description. The protocol for DNA extraction is found in the supplementary material of Ivanova et al. (2006). The primers LepF1, LepR1 (Hebert et al. 2004) and MLepF1, MLepR1 (Hajibabaei et al. 2006) were used by the CCDB. The protocol for COI amplification is found at http://dev.ccdb.ca/docs/CCDB_Amplification.pdf

### DNA sequencing

DNA extractions were performed following the method of Knölke et al. (2005) and with the NucleoSpin Tissue kit by Magerey-Nagel according to the manufacturer’s protocol. For alcohol preserved specimens, only tissue from the thorax was used for DNA extraction. For dried specimens, DNA was extracted from the abdomen or a leg, when the abdomen was missing.

The primers used are recorded in Table 4.

**Table 4.** Primers used for DNA sequencing. (F) stands for Forward primers, (R) for Reverse primers. The primers of the Nymphalidae Systematics Group can be found at http://nymphalidae.utu.fi/Nymphalidae/Molecular.htm

Origine	gene	Primers	References
mitochondrial	COI	HybLCO (F)	Wahlberg and Wheat 2008
		Nancy (R)	Wahlberg and Wheat 2008
		HybJerry (F)	Wahlberg and Wheat 2008
		HybPat (R)	Wahlberg and Wheat 2008
		K699 (R)	The Nymphalidae Systematics Group
		Ron (F)	The Nymphalidae Systematics Group
		Mila (R)	The Nymphalidae Systematics Group
		Brian (F)	The Nymphalidae Systematics Group
nuclear	wingless	HybLepWG1 (F)	Wahlberg and Wheat 2008
		HybLepWG2 (R)	Wahlberg and Wheat 2008
	EF-1α	Oscar-6143 (F)	Hundsdörfer et al. 2009
		Bosie-6144 (R)	Hundsdörfer et al. 2009

PCRs were performed using peqGOLD Taq DNA polymerase (PeqLab). In cases of weak or absent PCR result, a re-examination PCR was done using BIO-X-ACT Short Taq polymerase (Bioline). PCR protocols are given in Appendix II - Tables 1 and 2. Potential contamination was tested along with PCRs by control sample without DNA.

Success of gene amplification was evaluated by an electrophoresis with 1% agarose gel, subsequent gel dying with GelRed and analysis under ultraviolet light. PCR products were purified using ExoSAP-IT (USB Corporation).

Sequence PCR was done with the BigDye Terminator-Kit of Applied Biosystems. The amount of each product is reported in Appendix II - Table 3. The PCR programme is reported in the BigDye Terminator Sequencing Kit protocol. The sequences were obtained from the sample analysis on a 3130 Genetic Analyzer (Applied Biosystems).

### Sequence analyses

Sequence alignment was done with BIOEDIT 7.1.3 (Hall 1999) and PhyDE (Phylogenetic Data Editor) 0.9971 (Müller et al. 2011). Nucleotide positions that proved ambiguous in the pherogram were coded according to the IUPAC nucleotide ambiguity code. Depending on the quality of the sequences, the 5’ and 3’ ends of the sequences were deleted in cases of high error rates for the nucleotide assignment from the phenogram. We used the programme DAMBE (Xia and Xie 2001) to generate the molecular data with third codon position deleted. The alignement was exported as fasta, nexus and phylip files. Sequences were deposited on European Nucleotide Archive, and can be accessed via the following links (for the COI and the nuclear sequences respectively):

http://www.ebi.ac.uk/ena/data/view/HG793012-HG793020; http://www.ebi.ac.uk/ena/data/view/HG793012-HG793020

### Genetic distances

Genetic distances between barcoding sequences of COI are given in Table 5. Distances were calculated using DAMBE. The GTR model was used as the substitution model.

**Table 5.** Distance matrix between *Catharylla* species calculated with GTR correction. Values are given in %.

	*Catharylla bijuga* BC MTD 1839	*Catharylla bijuga* BC MTD 1840	*Catharylla chelicerata* LEP 1290	*Catharylla chelicerata* BC MTD 1703	*Catharylla chelicerata* BC MTD 1704	*Catharylla coronata* BC MTD 1890	*Catharylla mayrabonillae* LEP 1126	*Catharylla mayrabonillae* 07-SRNP-113921	*Catharylla paulella* LEP 965	*Catharylla serrabonita* LEP 979	*Catharylla serrabonita* BC MTD 1887	*Catharylla serrabonita* BC MTD 1843	*Catharylla tenellus* BC MTD 1842
*Catharylla bijuga* BC MTD 1840	5.05												
*Catharylla chelicerata* LEP 1290	16.61	14.63											
*Catharylla chelicerata* BC MTD 1703	16.84	14.83	0.62										
*Catharylla chelicerata* BC MTD 1704	16.63	14.63	0.46	0.15									
*Catharylla coronata* BC MTD 1890	11.55	10.61	13.34	13.35	13.14								
*Catharylla mayrabonillae* LEP 1126	15.55	15.8	16.6	16.83	16.61	11.22							
*Catharylla mayrabonillae* 07-SRNP-113921	14.82	15.07	15.63	15.63	15.42	9.79	4.34						
*Catharylla paulella* LEP 965	13.66	13.32	16.41	16.63	16.42	11.13	7.57	6.29					
*Catharylla serrabonita* LEP 979	10.16	9.75	13.46	13.46	13.46	8.48	14.58	13.11	12.59				
*Catharylla serrabonita* BC MTD 1887	11.1	10.41	15.06	14.65	14.86	7.74	14.64	13.68	13.57	3.24			
*Catharylla serrabonita* BC MTD 1843	10.37	9.7	14.48	14.07	14.28	7.59	14.49	13.11	13.01	2.21	0.77		
*Catharylla tenellus* BC MTD 1842	12.29	9.87	14.72	14.95	14.73	7.3	13.42	13.06	12.93	7.45	6.44	6.47	
*Catharylla tenellus* BC MTD 1709 1710 1888	11.8	10.52	15.01	15.24	15.03	7.31	13.15	12.08	12.14	6.72	7.17	6.49	3.34

### Phylogenetic analyses
Morphology-based analysis

The 21 characters are listed in Table 6. Characters 12, 16 and 17 were polarized into two sets of continuous values of ratios, with the limit selected subjectively where the gap between two groups of values appeared to be the largest. Phylogenetic analyses were run under PAUP 4.0b10 (Swofford 2002). A 12-taxa dataset was analysed, with *Crambus pascuella* and *Crambus uliginosellus* set as outgroups. Maximum parsimony analyses were performed using the Branch-and-bound method as searching algorithm with parameters left unchanged. The bootstrap resampling method with 1000 replicates was used. The resulting tree was a 50% majority-rule consensus tree with bootstrap (BS) values assigned to each node. BS supports are reported on the tree of Figure 42.

**Table 6.** Character matrix of *Catharylla* and related taxa. A question mark refers to an unknown state for character 21, or an uncoded state (*Crambus pascuella*, character 7).

**characters \ taxa**	*Catharylla bijuga*	*Catharylla chelicerata*	*Catharylla coronata*	*Catharylla gigantea*	*Catharylla mayrabonillae*	*Catharylla paulella*	*Catharylla serrabonita*	*Catharylla tenellus*	*Argyria lacteella*	*Micrelephas pictellus*	*Crambus pascuella*	*Crambus uliginosellus*
**1.** Labial palpi ringed with brown at 1/3 and 2/3: absent (0); present (1)	1	0	0	0	1	0	0	0	0	0	0	0
**2.** Length of labial palpi/eye diameter: >2/1 (0); < 2/1 (1)	1	1	1	1	1	1	1	1	1	1	0	0
**3.** Hindwing color: yellowish / creamy white (0); white (1)	1	1	0	1	1	1	0	0	1	0	0	0
**4.** Uncus in lateral view: horizontally straight (0); downcurved (1)	1	0	1	0	0	0	1	1	1	0	0	0
**5.** Dorsal furrow on uncus: absent (0); present (1)	0	0	1	0	0	0	1	1	0	0	0	0
**6.** Uncus dorsally setose (0); bare, or with few setae (1)	1	0	1	0	0	0	1	1	0	1	0	0
**7.** Uncus apex: unilobed, regularly rounded (0); regularly rounded with narrow tip (1); narrowing to a point (2); apically slightly bifid (3)	2	1	3	1	2	2	3	3	2	0	?	1
**8.** Apex of valva: narrowed or rounded (0); quadrangular, truncated (1)	0	1	0	1	0	0	0	0	0	0	0	0
**9.** Gnathos: almost straight with apex pointing upward (0); bent of about 90° angle (1); regularly curved (2)	0	2	0	2	1	1	0	0	1	0	0	0
**10.** Transtilla: absent (0); present (1)	0	0	1	0	0	0	1	1	0	0	0	0
**11.** Lateroventral projections on juxta: absent (0); present (1)	1	0	1	0	1	1	1	1	0	0	0	0
**12.** Ratio of dorsal roof of tegumen on uncus length: < 1/2 (0); > 1/2 (1)	0	0	0	0	1	1	0	0	0	0	1	1
**13.** Latero-basal tuft of hairs on uncus: absent (0); present (1)	0	1	1	1	0	0	1	1	1	1	0	0
**14.** Vesica: with one cornutus (0); with crest of cornuti (1); without cornuti (2)	0	1	1	1	0	0	2	2	1	2	2	0
**15.** Ventral shape of papillae anales in lateral view: not produced (0); slightly produced (1)	1	1	0	1	1	1	0	0	1	0	0	0
**16.** Ratio of anterior apophyses/papillae anales: > 0.1 (0); < 0.1 (1)	1	1	1	1	1	1	1	1	0	0	1	1
**17.** Ratio of posterior apophyses/papillae anales: > 0.5 (0); < 0.5 (1)	1	1	1	1	1	1	1	1	0	0	0	0
**18.** Ventral tongue shaped pronounced sclerotization postero-ventrally on ductus bursae: absent (0); present (1)	1	1	0	1	0	1	0	0	0	0	0	0
**19.** Ventral membrane of segment VIII: without tiny setae (0); with tiny setae (1)	1	1	1	1	0	0	1	1	0	0	0	0
**20.** Postvaginal sterigma: present (0); absent (1)	0	0	1	0	0	0	1	1	0	0	0	0
**21.** Forewing R4: free (0); stalked with R2+3 (1)	?	1	?	?	1	?	1	?	0	0	?	?

### Molecular-based analysis

Because of the age of some of the material used, only the COI barcode sequences were available for *Catharylla bijuga* and *Catharylla coronata*, and the whole COI gene for *Catharylla paulella*. For *Catharylla bijuga*, we used the sequence BC MTD 1840 to build the datasets as this sequence performed better than BC MTD 1839 in phylogenetic analyses. The COI sequence of LEP 972 was combined with that of wingless for LEP 973 given that the two samples come from the same population and are genetically very similar as attest the barcode sequences. We generated four different datasets from the sequence data: a complete dataset with all three genes sequences available for the 12 taxa (mol_1), a 12-taxa dataset with the 3^rd^ codon position of COI deleted (mol_2), a dataset restricted to the 7 taxa for which the sequence of the COI gene and at least of one nuclear gene were available (nucl_1), and the same data as nucl_1 but with the 3^rd^ codon position of the COI deleted (nucl_2). We used the programme RAxML (Stamatakis 2006) to perform phylogenetic analyses of these four datasets under the Maximum Likelihood algorithm. The analyses were run on the online platform CIPRES (Miller et al. 2010) with the web-server adapted interface of RAxML (Stamatakis et al. 2008). The version 7.2.8 of RAxML with the Black Box were used. As in the morphology-based analysis, *Crambus pascuella* and *Crambus uliginosellus* were set as outgroups in the analyses of mol_1 and mol_2, and *Crambus uliginosellus* was set as outgroup in the analyses of nucl_1 and nucl_2. For each dataset, three analyses were performed: one without partition, one with the data partitioned into the three genes, and one with the data partitioned into the three genes and the three codon positions in each gene. According to Mr-AIC (Nylander 2004), the model which best fits our data is GTR+I+G, hence this model was used as evolution model, with parameters estimated for each partition, and the proportion of invariable sites estimated. Bootstrapping was automatically halted by RAxML when a sufficient number of bootstrap replicates was reached. The number of bootstrap replicates is indicated for each analysis. The resulting trees were 50% majority-rule consensus trees with bootstrap values assigned to each node. BS supports of each analysis are reported on the tree (Fig. 42).

### Combined bayesian analysis

A nexus file was created for the 12 taxa investigated, with the four following partitions: (1) the morpho-matrix (Table 6), (2) COI, (3) wingless, (4) EF-1α, and was analysed using Mr-Bayes 3.2.1 (Huelsenbeck and Ronquist 2001). The settings were set as follows to fit to the GTR+I+G model: number of states (“nst”) = 6, rate variation among sites (“rates”) = gamma-shaped rate variation with a proportion of invariable sites (“invgamma”). The relative rates of substitution (“revmat”), the transition/transversion rate ratio (“tratio”), the stationary nucleotide frequencies (“statefreq”), and the alpha shape parameter of the gamma distribution (“shape”) were unlinked in order to allow them to vary among partitions. The number of generations was set to 3,000,000. *Crambus uliginosellus* was chosen as outgroup. The results were considered good when the standard deviation of split frequencies between the two independent runs was below 0.01. Nodes with a posterior probability (PP) over 0.95 are considered well supported. PP values are reported on the tree (Fig. 42).

## Results

### Catharylla

Zeller, 1863

http://species-id.net/wiki/Catharylla

Catharylla Zeller 1863: 50, Bleszynski and Collins 1962: 226, Landry 1993: 1088, Munroe 1995: 35, Nuss et al. 2013.

#### Type species.

*Crambus tenellus* Zeller, 1839, by subsequent designation by Schaus 1922: 131.

#### Diagnosis.

*Catharylla* species have snow white to creamy white wings and short labial palpi. They can be separated from other Argyriini by the presence on the forewing of median and subterminal thin transverse lines, slightly curved, convex on costal 1/3. The labial palpi are also shorter in comparison to those of *Vaxi*. The highly variable male genitalia do not show any synapomorphy or generic diagnostic character. In females, a possible synapomorphy is the strongly reduced anterior and posterior apophyses of abdominal segments VIII and IX, but this is shared with some Crambini and a few other Crambinae (see Landry 1995).

#### Redescription.

**Figures 1–8.**
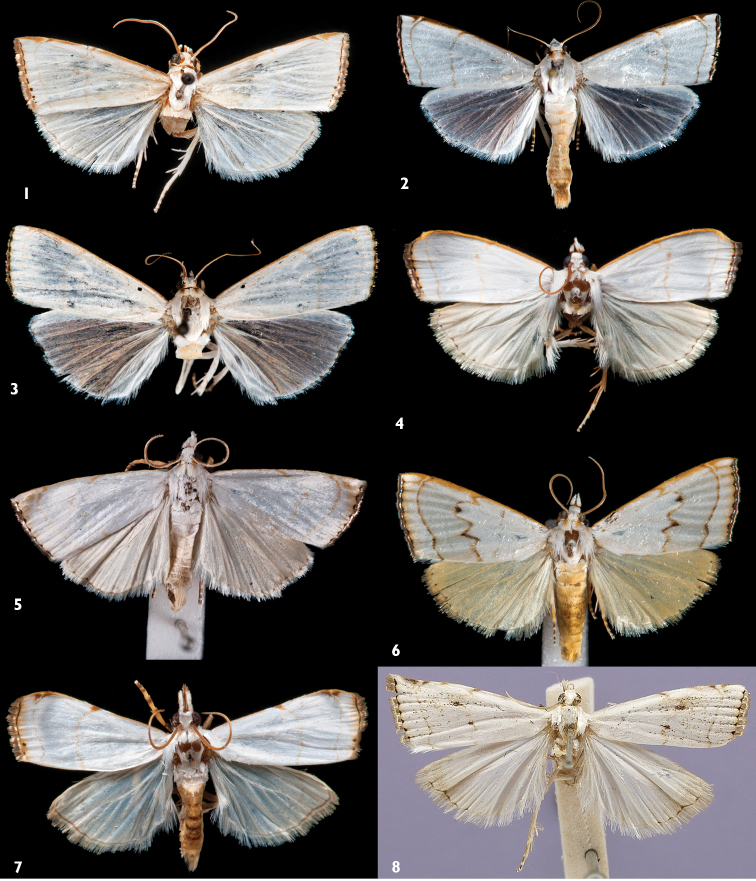
Habitus of *Catharylla* species: **1** *Catharylla bijuga* sp. n., holotype, French Guiana, Pied Saut, Oyapock River (CMNH) **2** *Catharylla chelicerata* sp. n., Parcelles CIRAD de Combi, plantations expérimentales pk 1.8, 5°18'N, 52°55'30"W (MHNG) **3** *Catharylla gigantea* sp. n., holotype, Brazil, Amazonas, Manaus, Reserva Ducke, AM-010, km 26, 2°55'S, 59°59'W **4** *Catharylla tenellus* Zeller, Brazil, Saõ Paulo, Bertioga (Becker Coll.) **5** *Catharylla coronata* sp. n., holotype, Brazil, Espirito Santo, Linhares (USNM) **6** *Catharylla serrabonita* sp. n., holotype, Brazil, Bahia. Camacan, Reserva Serra Bonita, 15°23'S, 39°33'W, 800m (Becker Coll.) **7** *Catharylla mayrabonillae* sp. n., holotype, Ecuador, Napo, Misahualli (Becker Coll.) **8** *Catharylla paulella* Schaus, holotype, Brazil, Sao Paulo state, Sao Paulo (USNM).

**Figure 9.**
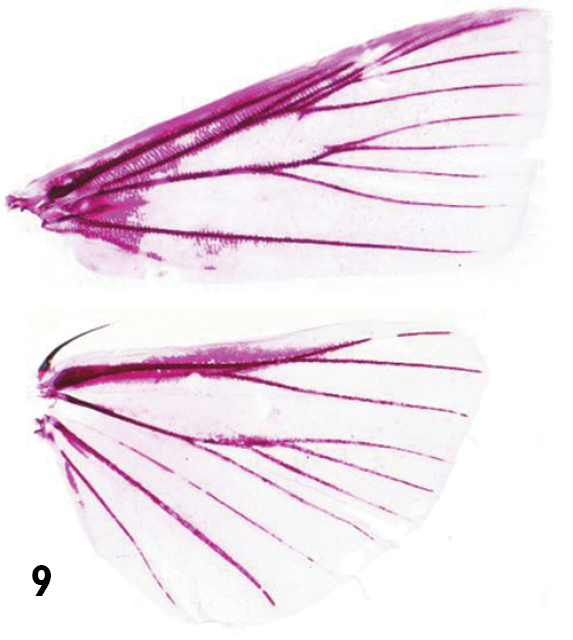
Male wing venation of *Catharylla* species: *Catharylla chelicerata* sp. n., paratype ♂, French Guiana, Roura, Camp Patawa; slide MHNG 6272 (MHNG).

Head white, chaetosemata present. Antenna brown, covered with light ochreous to brown scales. Maxillary palpus light ochreous to brown, white tipped. Labial palpus 1.0–1.95 × width of head, curved upward; white basally, light ochreous to ochreous, white tipped, with some brown or dark brown. Thorax white, with ochreous to brown scales at collar. Foreleg coxa white to whitish brown, femur dorsally brown to dark brown, tibia and tarsomeres distally ringed with dark brown. Midleg white to light ochreous with tibia-femur joint ashen brown, with pair of spurs at apex of tibia, tarsomeres II–V dorsally brown to dark brown, with white tips. Hindleg white, with 2 pairs of spurs on tibia, tarsomeres as on midleg. Male frenulum simple, frenulum hook present; female frenulum with 3 or 4 acanthae. Forewing length: 7.5–15 mm in males; 9.5–22 mm in females. Wing venation (of *Catharylla chelicerata*) (Fig. 9): R1 present and free, not connected to Sc; R2 free; R3 connected with R4 at 3/4; R5 stalked with R3+R4 at 1/4; M1 from upper corner of cell; cell opened between M1 and M2; M2 and M3 not stalked; CuA1 from lower corner of cell; CuA2 at distal 1/3 of cell; 1A+2A strong. Hindwing Sc+R1 connected to Rs at distal 1/3; M1 connected to Sc+R1 by short narrow vein; M3 connecting to M2 at distal 1/3, CuA1 connecting to M2 at half of length and CuA2 connecting at basal 2/5; 1A unforked; 2A unforked, strong; 3A present, unforked. Forewing (Figs 1–8) background snow white; pattern with costal margin ochreous to brown, sometimes faded; median and subterminal transverse lines thin, ochreous to brown, convex toward costa; outer margin ochreous, sometimes with dark brown spots between veins, or spots forming a continuous line; fringes white; verso light ochreous to ochreous; with marginal spots pronounced. Hindwing snow white to cream-colored; with hairs along 2A and root of M2; with small dark brown spots on outer margin, sometimes in continuous line; sometimes with postmedian transverse line; verso white with marginal spots pronounced.

**Figures 10.**
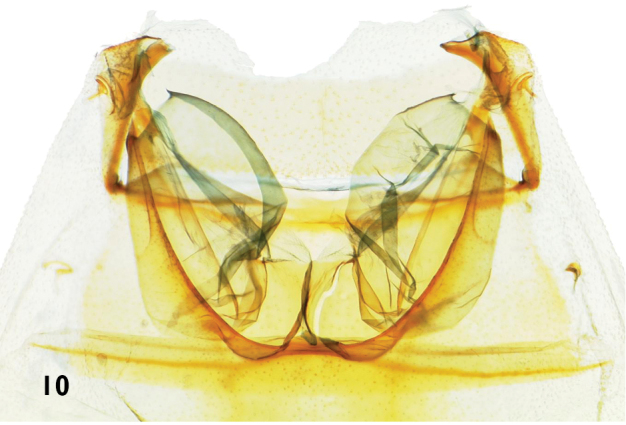
Tympanal organs of *Catharylla* species: *Catharylla paulella* Schaus, ♀, Brazil, Distrito Federal, Planaltina, slide BL 1751 (Becker Coll.).

Tympanal organs (Fig. 10): Transverse ridge regularly rounded. Tympanic pockets more or less extending beyond transverse ridge. Tympanic bridge present, straight, lightly sclerotized. Tympanic drum more or less ovoid.

**Figures 11–16.**
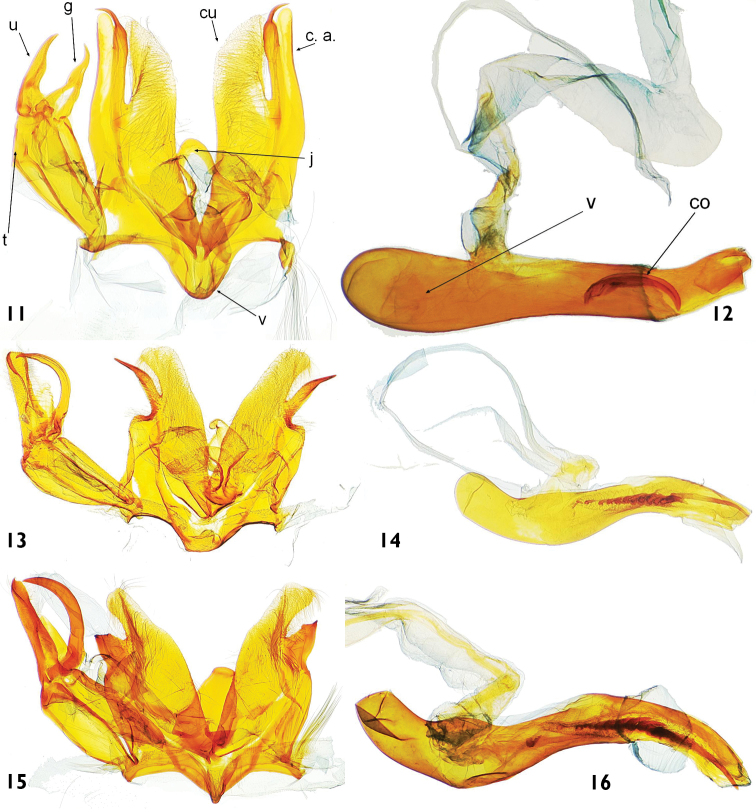
Male genitalia features of *Catharylla* species: **11–12** *Catharylla bijuga* sp. n., paratype, French Guyana, St-Jean-de-Maroni, slide BL 1719 ♂ (BMNH) **11** Genitalia without phallus. cu: cucculus; c. a.: costal arm; g: gnathos; j: juxta; t: tegumen; u: uncus; v: vinculum **12** Phallus in lateral view. v: vesica; c: cornutus **13–14** *Catharylla chelicerata* sp. n., paratype, Brazil, Amazonas, Rio Negro, Mirapinima, slide BL 1714 ♂ (MHNG) **13** Genitalia without phallus **14** Phallus in lateral view **15–16** *Catharylla gigantea* sp. n., holotype, Brazil, Amazonas, Reserva Ducke, slide BL 1747 ♂ (BMNH) **15** Genitalia without phallus. 1 **6** Phallus in lateral view.

**Figures 17–22.**
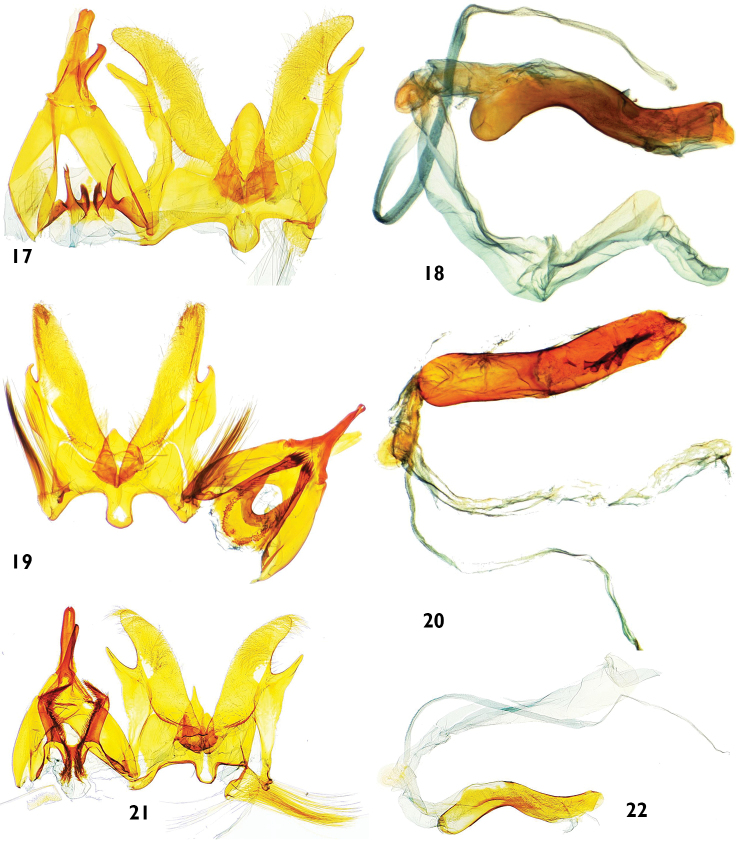
Male genitalia features of *Catharylla* species: **17–18** *Catharylla tenellus* Zeller **17** Genitalia without phallus, Brazil, Minas Gerais, Caraça, slide BL 1755 ♂ (Becker Coll.) **18** Phallus in lateral view, Brazil, Saõ Paulo, Bertioga, slide BL 1746 ♂ (USNM) **19–20** *Catharylla coronata* sp. n. paratype, Brazil, Paranà, Rio Negro, slide BL 1730 ♂ (ISZP) **19** Genitalia without phallus **20** Phallus in ventral view **21–22** *Catharylla serrabonita* sp. n. paratype, Brazil, Bahia, Camacan, Serra Bonita Reserve, slide BL 1776 ♂ (MHNG) **21** Genitalia without phallus **22** Phallus in lateral view.

**Figures 23–29.**
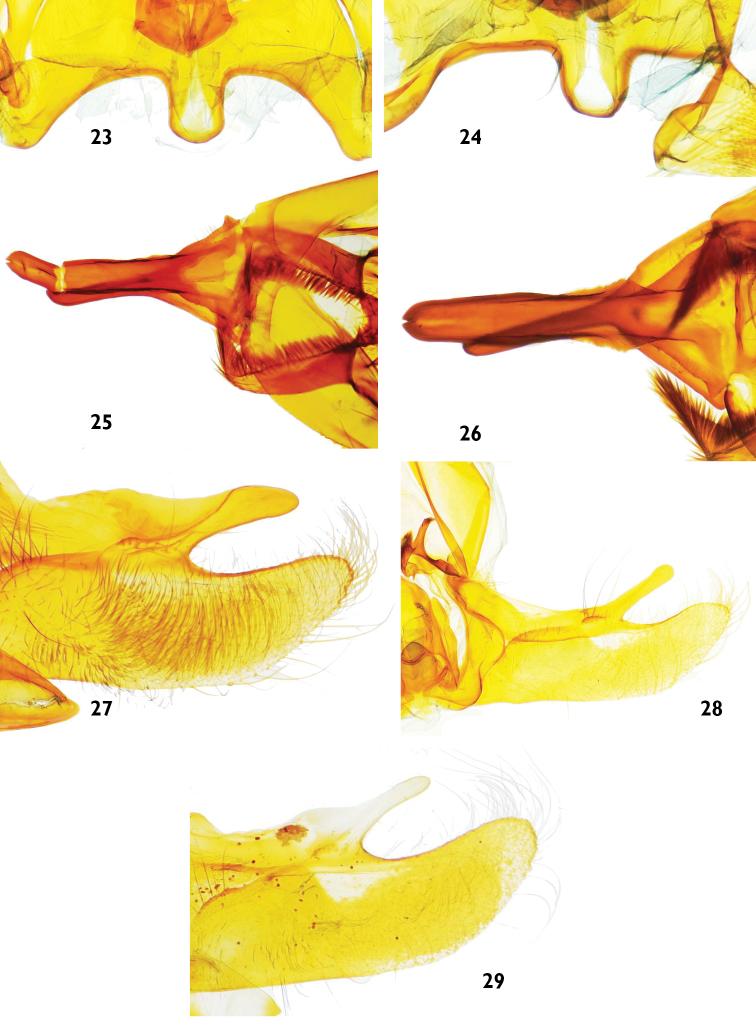
Male genitalia features of *Catharylla* species: **23–24** Vinculum of *Catharylla serrabonita* sp. n. **23** Brazil, Espírito Santo, Linhares, slide BL 1745 ♂ (USNM) **24** Brazil, Bahia, Camacan, Serra Bonita Reserve, slide BL 1776 ♂ (MHNG) **25–26** Uncus of *Catharylla serrabonita* sp. n. **25** Brazil, Espírito Santo, Linhares, slide BL 1745 ♂ (USNM) **26** Brazil, Bahia, Camacan, Serra Bonita Reserve, slide BL 1776 ♂ (MHNG) **27–29** Costal arm of *Catharylla tenellus* **27** São Paulo, Ubatuba, Picinguaba, slide BL 1757 ♂ **28** Minas Gerais, Caraça, slide BL 1746 ♂ **29** Brazil, Bahia, Porto Seguro, A. d’Ajuda, slide TL 9 ♂.

**Figure 30–33.**
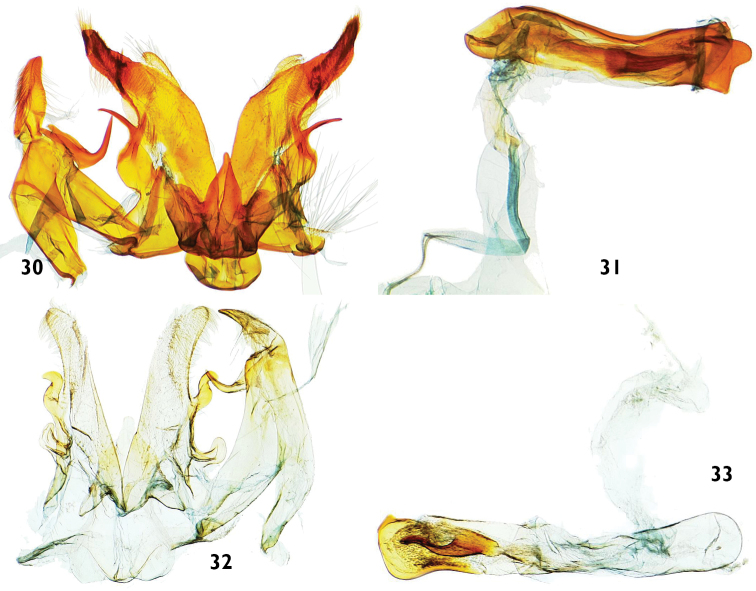
Male genitalia features of *Catharylla* species. **30–31** *Catharylla mayrabonillae* sp. n., paratype, Peru, Agnaytia, Huallaga, 400 m, slide BL 1724 ♂ (CNC) **30** Genitalia without phallus **31** Phallus in lateral view **32–33** *Catharylla paulella* Schaus, Bolivia, provincia del Sara, slide GS-6682-SB (BMNH) **32** Genitalia without phallus **33** Phallus in lateral view.

Male genitalia (Figs 11–33): Uncus long, basally wide, straight or downcurved, bare or setose. Gnathos arms connecting at 1/4 to 1/2 from base, thin, slightly curved to hook shaped, with apex pointing upward. Tegumen arms enlarging toward uncus, connecting dorsally. Valva with cucculus long, densely setose on inner side, apically more or less rounded, slightly curved upward; costal arm present, variable in shape. Transtilla present only in *Catharylla tenellus* group, strongly developed. Juxta medially curved downward, narrowing toward rounded apex, slightly directed downward apically, basally triangular, sometimes with additional lobes at base or ventro-lateral projections. Vinculum of medium width; saccus short, rounded, directed anterad and slightly upward. Phallus straight or curved, usually more strongly sclerotized at apex; vesica without cornuti, with one cornutus, or with crest of cornuti.

**Figures 34–41.**
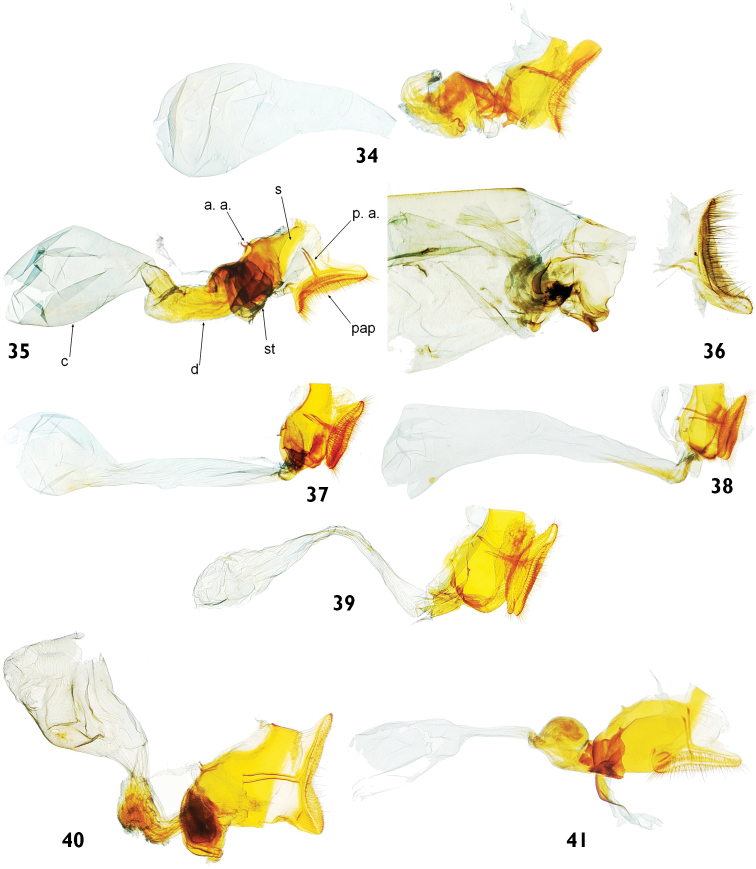
Female genitalia of *Catharylla* species. **34** *Catharylla bijuga* sp. n. in lateral view, paratype, Suriname, Sipaliwini District, Thibiti area, Kabo-Creek, slide BL 1732 ♀ (Schouten Coll.) **35** *Catharylla chelicerata* sp. n., paratype, Brazil, Reserva Ducke, slide BL 1711 ♀ (CNC); pap: papillae anales; p. a.: posterior apophyses; s: segment VIII; a. a.: anterior apophyses; st: sterigma; d: ductus bursae; c: corpus bursae **36** *Catharylla gigantea* sp. n. paratype, French Guyana, Saint-Jean-du-Maroni, slide n°6679SB (BMNH) **37** *Catharylla tenellus* Zeller, Brazil, Rio de Janeiro, slide BL 1733 ♀ (CMNH) **38** *Catharylla coronata* sp. n. paratype, Brazil, Paranà, Curitiba, slide BL 1753 ♀ (Becker Coll.) **39** *Catharylla serrabonita* sp. n. paratype, Brazil, Espírito Santo, Linhares, slide BL 1759 ♀ (Becker Coll.) **40** *Catharylla mayrabonillae* sp. n. paratype, French Guyana, Saint-Jean-du-Maroni, slide BL 1720 ♀ (BMNH) **41** *Catharylla paulella* Schaus, Brazil, Distrito Federal, Planaltina, slide BL 1751 ♀ (Becker Coll.).

Female genitalia (Figs 34–41): Papillae anales strongly setose, connecting dorsally and ventrally, usually slightly produced dorsally, with basal band of sclerotization. Posterior apophyses 0.25–0.45 × length of papillae anales, straight, regularly thin. Tergite VIII narrow, with postero-dorsal line of setae. Anterior apophyses reduced, 0.01–0.1 × length of papillae anales. Sternite VIII about twice length of tergite, not connecting ventrally in *tenellus* species group. Sterigma present, strongly sclerotized except in *tenellus* species group, usually forming pockets of variable shape; reduced to sclerotized lamella antevaginalis in *Catharylla bijuga*. Ductus seminalis connecting posteriorly at base of ductus bursae. Ductus bursae long, at least 2 × length of corpus, wide, basally curved. Corpus bursae usually rounded, egg-shaped, often enlarging progressively from ductus bursae, usually with one signum, sometimes without; corpus and ductus bursae covered with minute spicules.

**Figure 42.**
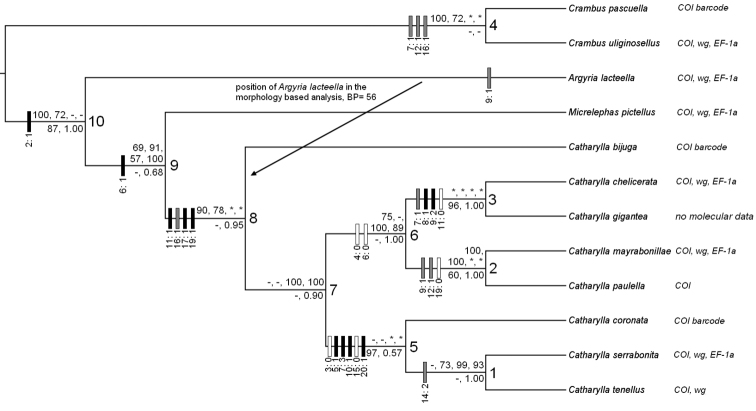
50% majority-rule consensus tree of the combined Bayesian analysis of the morphological and molecular datasets combined with 3'000'000 generations. Values above branches are the respective BS supports of the phylogenetic analyses with Maximum Likelihood algorithm of mol_1 (gene + codon partition, 800 BS replicates), mol_2 (gene partition, 450 BS replicates), nucl_1 (gene partition, 150 BS replicates), and nucl_2 (gene partition, 150 BS replicates). Values below branches are the BS supports of the phylogenetic analysis of the morphological data and the PP of the combined Bayesian analysis. “-” represent values under the majority rule; “*” represent the absence of value because of the absence of one or several taxa in the considered analysis. Boxes upon branches represent character changes: black boxes represent unique transformations to the apomorphic state; grey boxes represent multiple transformations to the apomorphic state; white boxes represent reversals to the plesiomorphic state.

#### Distribution.

**Figure 43.**
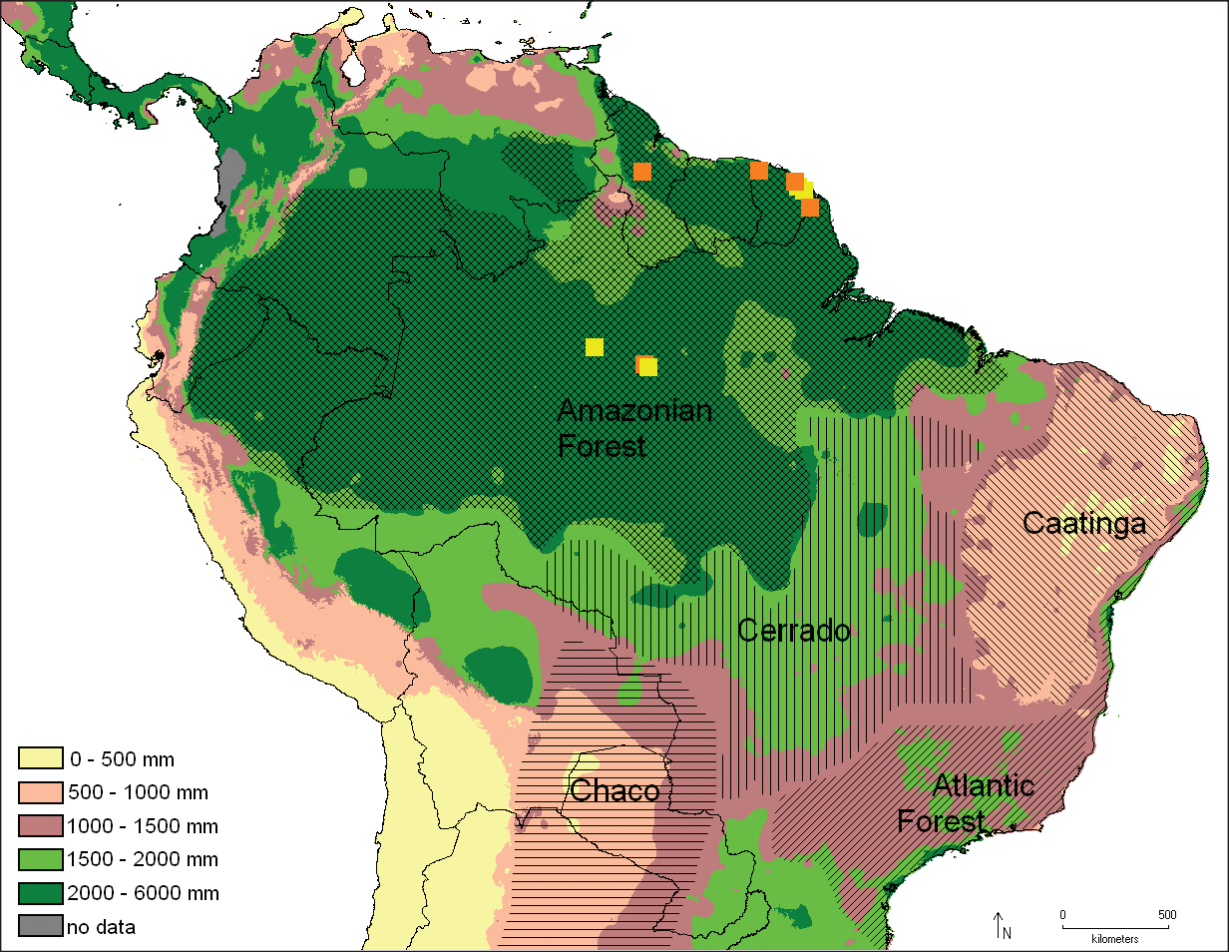
Distribution of *Catharylla chelicerata* (yellow) and *Catharylla gigantea* (orange) with the pluviometry and the biomes reported. Distribution of the biomes taken from Vanzolini and Heyer (1987: map n°3).

**Figure 44.**
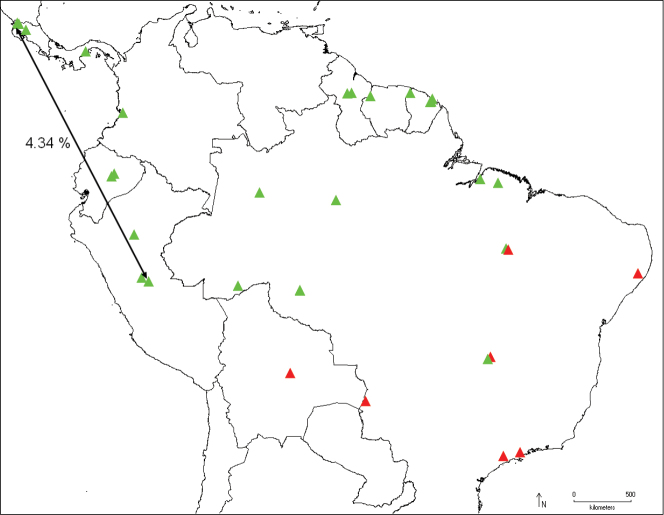
Distribution of *Catharylla mayrabonillae* (green) and *Catharylla paulella* (red), and distance between barcode haplotypes 07-SRNP-113921 and LEP 1226 (*Catharylla mayrabonillae*).

**Figure 45.**
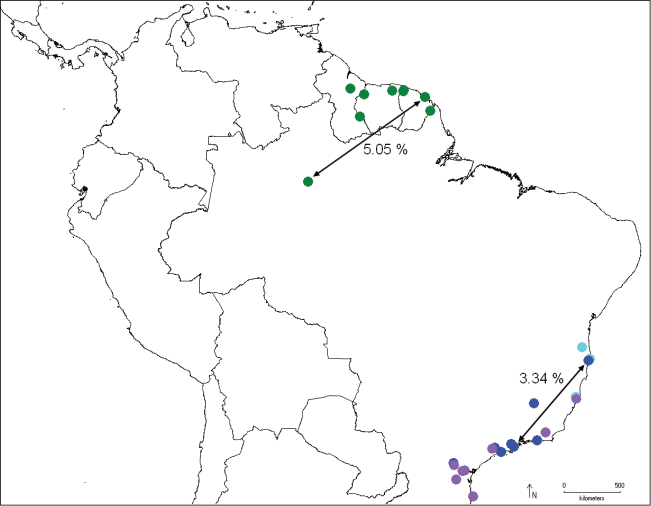
Distribution of *Catharylla bijuga* (green), *Catharylla coronata* (light purple), *Catharylla serrabonita* (sky blue) and *Catharylla tenellus* (blue), and distances between barcode haplotypes BC MTD 01839 and BC MTD 1840 (*Catharylla bijuga*), and BC MTD 1709 and BC MTD 1842 (*Catharylla tenellus*).

**Figure 46.**
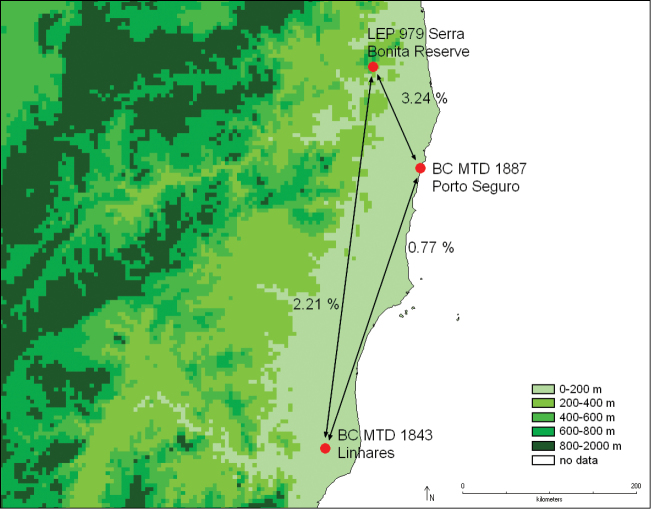
Distribution of *Catharylla serrabonita* and percentage of nucleotide differences in barcode sequences between different samples.

The genus is restricted to the Neotropical Region, from Costa Rica to Santa Catarina, Brazil, from sea level to 1300 m (Figs 43–46).

#### Biology.

The biology of the species remains unknown. In the Serra Bonita Reserve in march 2011, we observed *Catharylla serrabonita* in its environment, i.e. forested hills up to about 950 m in elevation, surrounded by cacao or coffee plantations in the lowlands. The moths were coming to light, usually very late (after 23:00).

#### Phylogenetic relationships and monophyly.

Presumably, given the reduced labial palpi and the forewing color and pattern, *Catharylla* has been placed in the Argyriini (Munroe 1995). But our phylogenetic analyses do not support tribe Argyriini with *Catharylla* included, and the genus seems to be most closely related to *Micrelephas*.

### Catharylla bijuga

T. Léger & B. Landry sp. n.

http://zoobank.org/9FC3D8A6-A172-443C-A52A-BE510976DD0A

http://species-id.net/wiki/Catharylla_bijuga

Figs 1 , 11 , 12 , 34 , 45

#### Type material.

**Holotype.** ♂, with labels as follows: “Pied Saut, | Oyapok [sic] River, | French Guiana, | S. M. Klages | C. M. Acc. 6111.”; “Dec[ember]. | 1917”; “HOLOTYPE | Catharylla bijuga | T. Léger & B. Landry” [red label]; “Catharylla | ramona sp. n. | det. Bleszynski, 1969”; “MANUSCRIPT | NAME” [white card with red lettering and thin red rectangle submarginally]; “BL 1744 ♂”. Deposited in CMNH.

**Paratypes.** 16 ♂, 9 ♀. BRAZIL: 1 ♂ (genitalia on slide BL 1748, used for DNA Barcoding BC MTD 01840), Amazonas, P[ar]q.[ue] Nac.[ional] do Jaú, Rio Jaú, bg. Miratucú, 1°57'S, / 61°49'W, 26–27.vii.1995, U[ltra] V[iolet] light sheet (R. W. Hutchings) (USNM). FRENCH GUIANA: 2 ♂ (1 with genitalia on slide Pyralidae Brit. Mus. slide N°15893) with same data as holotype (BMNH); 5 ♂, 1 ♀ (2 ♂ with genitalia on slides Pyralidae Brit. Mus. Slide N°15891, N°15892, ♀ with genitalia on slide BL 1735) with same locality as holotype except i.1918 (1 ♂), ii.1918 (4 ♂, 1 ♀) (S. M. Klages) (BMNH, CMNH); 1 ♂, 2 ♀, Parcelles CIRAD de Combi, plantations expérimentales pk 1.5, 5°18'N, 52°55'30 W, 4.iii.2011, piège lumineux [light trap] (B. Hermier) (♂ with Hermier n° 24340, 2 ♀ with labels Hermier n° 24341 & 24345) (MHNG); 3 ♂, 2 ♀ (2 ♂ with genitalia on slides BL 1719 and Pyralidae Brit. Mus. Slide N° 7815, 2 ♀ with genitalia on slides BL 1739 and BL 1740), Saint-Jean-du-Maroni (Le Moult) (BMNH); 1 ♂, Saint-Laurent-du-Maroni (USNM); 1 ♂ (genitalia on slide BL 1694, (used for DNA barcoding BC MTD 01839) Roura, 3.6km E[ast] Roura at r[oa]d to Crique Gabrielle, 50m, 20.iv.1994, at light (J. S. Miller & C. Snyder) (AMNH); 1 ♀ (genitalia on slide BL 1734), Cayenne, iii.1917 (CMNH). GUYANA: 1 ♂, New River 1938 (C.A. Hudson) (BMNH); 1 ♂, Mallali [sic] (USNM). SURINAME: 1 ♀ (genitalia on slide USNM 52888), Geldersland, Surinam River (USNM); 1 ♀ (genitalia on slide BL 1732), Sipaliwini Distr[ict], Thibiti area, Kabo Creek, partly swampy, primary forest on hilly slopes, ca. 2km from river, 29.v.1989 (J. Beerlink) (Schouten Coll.).

COI barcode sequence of paratype BC MTD 01839 (654 bp): ACATTATATTTTATCTTCGGAATTTGAGCAGGAATAGTTGGAACATCCCTAAGACTTTTAATTCGAGCAGAATTAGGTAATCCAGGTTCTCTTATTGGTGACGACCAAATTTATAATACTATTGTTACTGCTCATGCATTTATTATAATTTTTTTTATAGTTATGCCAATTATAATTGGAGGATTCGGTAATTGATTAGTTCCATTAATATTGGGAGCACCAGATATAGCATTCCCACGAATAAATAATATAAGATTTTGATTACTCCCCCCCTCTTTAATCCTATTAATTTCTAGAAGAGTTGTAGAAAATGGAGCTGGAACAGGATGAACAGTTTACCCCCCACTTTCATCAAATATTGCTCATAGTGGTAGATCTGTAGATTTAGCAATTTTTTCTCTACACTTAGCAGGAATTTCATCAATCTTAGGAGCTATTAATTTTATTACAACAATTCTTAATATACGAATTAATGGTTTATCTTTCGATCAAATACCTTTATTTGTTTGATCTGTAGGAATTACAGCTTTACTTCTTCTCTTATCCTTACCCGTATTAGCTGGTGCTATTACTATACTTTTAACTGATCGAAATTTAAATACATCTTTTTTTGATCCTGCTGGAGGAGGAGATCCTATCCTTTACCAACACTTA

#### Diagnosis.

On the forewing (Fig. 1), the seven, thin, marginal dark brown dashes with the most tornal two shaped like spots will separate this species from the others. In male genitalia (Fig. 11), the strongly sclerotized double costal arm of the valva with the ventral arm tubular is a distinctive character. In female genitalia, the best diagnostic character is the sclerotized projection latero-ventrally on sternite VIII (Fig. 34).

#### Description.

Male (n = 17) (Fig. 1): Antenna brown with light ochreous scales; with patch of dark brown scales at base. Maxillary palpus ringed with brown at base and half of length, white tipped. Labial palpus: 1.4–1.6 mm long; ochreous, slightly lighter basally, ringed with dark brown at 2/3, white tipped. Thorax slightly ochreous at collar. Foreleg coxa white; femur ochreous, dark brown dorsally; tibia and tarsomeres ochreous, distally ringed with brown. Midleg and hindleg light ochreous; tibia-femur joint brown on midleg; tarsomeres II–V brown to dark brown on upperside, with white ringed tips. Abdomen dull white to greyish brown. Forewing length: 9.0–10.5 mm; snow white, with yellow ochreous to brown costal margin, partially disrupted when meeting transverse lines; median line yellow ochreous; subterminal line yellow ochreous to brown, forming small triangular spot on costal margin; subapical triangle on costal margin ochreous; outer margin slightly ochreous with five dark brown dashes regularly spaced or sometimes forming faintly continuous line, and one cubital and one anal spots, with cubital spot slightly displaced toward base; fringes brass colored; underside dull white to light ochreous along costal margin, with marginal dashes pronounced. Hindwing snow white, veins slightly ochreous, with shiny aspect; marginal line thin, brown, pronounced up to CuA1, then shiny white; fringes white; underside white, with same margin as on recto.

Tympanal organs (n = 8): Tympanic pockets extending slightly beyond transverse ridge, rounded. Tympanic drum elongate, more or less oval, postero-laterally extended beyond transverse ridge.

Male genitalia (n = 8) (Figs 11, 12): Uncus slightly down-curved, about 3/5 length of tegumen arms, with few setae laterally; tip pointed; uncus arms not separated at base, forming low bump medio-ventrally. Gnathos arms joining at half their length; distal half with short, rounded, dorsal projection at base; directed upward subapically at about 50° angle; slightly shorter than uncus and thinner. Tegumen pedunculi progressively widening toward uncus; dorsal connection of tegumen about 1/3 length of pedunculi; ventral margin straight; dorsal margin slightly convex, bare. Cucculus moderately wide, narrowing in distal 1/4; costal arm of valva double, bare, about as long as cucculus, joined to cucculus until 3/5 of its length; ventral arm thin, tubular, strongly sclerotized, slightly curved inward, apex directed upward, narrowed, pointed; dorsal arm broader, slightly shorter than ventral arm, straight, apically rounded, less thickly sclerotized than ventral arm. Vinculum enlarging latero-dorsally, ventrally narrow; saccus short, rounded. Juxta triangular, apically broadly rounded, slightly curved downward, basally projected into two large lateral lobes. Phallus almost straight, with slightly upturned sclerotized apex; vesica covered with microspicules barely visible, with one large, curved, pointed cornutus.

Female (n = 10): Labial palpi: 1.1–1.4 mm long. Forewing length: 11–15 mm; frenulum triple.

Female genitalia (n = 6) (Fig. 34): Papillae anales slightly projected ventrally and dorsally, dorsally forming prominent sclerotized rounded bulge. Posterior apophyses widened basally, 0.35–0.5 × length of papillae anales. Segment VIII circular in cross section, enlarging progressively toward papillae. Tergite VIII narrow, about 2/5 length of sternite VIII, with short setae along posterior edge. Anterior apophyses wide at base, about 0.1 × length of papillae. Sterigma with thin slightly sclerotized membrane covered with minute spicules dorsad of ostium bursae, with posterior margin slightly indented; with sclerotized projection laterad from sternite VIII antero-ventrally, with tip bifid, longer part directed downward, shorter part lateral, curved posterad. Basal part of ductus bursae ventrally sclerotized, looping and narrow, progressively widening toward corpus bursae. Corpus bursae poorly differenciated from ductus, twice as long as wide, without signum.

#### Distribution.

The species occurs in lowlands in the three Guianas and Brazil (Fig. 45).

#### Etymology.

Bijuga comes from the Latin *bijugus, a, um* which means “yoked together, double”, in reference to the bifid costal arm of the male genitalia.

#### Notes.

In some paratypes from French Guiana the collecting data mention a “pk” (=”point kilométrique”). This kilometric marker refers to the distance of the collecting spot on the forest road to the nearest main road. CIRAD (Centre de coopération internationale en recherche agronomique pour le développement) refers to the name of the research institution leading agronomical research on the Combi site. the Combi site. When S. Bleszynski looked into *Catharylla*, he gave the manuscript name *Catharylla ramona* to this species, but never published it. The comparison of the tip of the tubular costal arm of the male genitalia and the female lateral projections of sternite VIII shows rather nicely that the male hooks the female genitalia during the mating process. The specimen collected in Parque Nacional do Jaú, Brazil, shows a divergence in COI barcode sequence of 5.05% with that of Roura, French Guiana. In morphology we find no significant difference corroborating this divergence. The relationships of this species to the others remain uncertain in our phylogenetic analyses.

### *Chelicerata* species group

**Diagnosis.** The synapomorphies of the group are the quadrangular valva with a truncated apex and the hook-shaped gnathos in the male genitalia. The *chelicerata* species group can be separated from the other *Catharylla* species based on additional diagnostic characters. Externally, the forewing has a clear, dark brown costal band, and its length is usually over 14 mm. In male genitalia, the apex of the uncus is regularly rounded with a short, narrow projection medially and the vesica shows one large, curved, pointed cornutus, preceded by a string of 13–14 smaller cornuti increasing in size toward apex. In female genitalia, the ductus bursae shows a pronounced, tongue-shaped projection postero-ventrally.

**Notes.** This group includes two species. The phylogenetic analyses restricted to the nuclear genes and the combined Bayesian analysis place the group as sister-group of the *mayrabonillae* species group (Fig. 42).

### Catharylla chelicerata

T. Léger & B. Landry sp. n.

http://zoobank.org/10B2350D-F0E5-4E09-BDE8-CE7D3F58A33F

http://species-id.net/wiki/Catharylla_chelicerata

Figs 2 , 9 , 13 , 14 , 35 , 43

#### Type material.

**Holotype.** ♂, with labels as follows: “/600/ Parcelles CIRAD de Combi, | plantations expérimentales pk 1,8 | 5°18'N, 52°55'30"W | 3.XII.2010 | B[ernard]. Hermier | [piège lumineux]”; 1 ♂, “Hermier | n° 23939”; “MHNG | ENTO ♂ | 00007213”; “Don de Bernard | Hermier | MHNG 2013”; “HOLOTYPE | Catharylla | chelicerata | T. Léger & B. Landry” [red label]. Deposited in MHNG.

**Paratypes.** 20 ♂, 4 ♀. BRAZIL: 1 ♂ (genitalia on slide BL 1714), Amazonas, Rio Negro, Mirapinima, 8.iv.1972 (E. G., I. & E. A. Munroe) (CNC); 9 ♂, 1 ♀, Reserva Ducke, km 26 Manaus–Itacoatiara Highway, 15.iv.1972 (1 ♂), 18.iv.1972 (1 ♂, with genitalia on slide BL 1721), 21.iv.1972 (2 ♂, one with genitalia on slide BL 1709), 16.v.1972 (2 ♂), 17.v.1972 (2 ♂, one with genitalia on slide BL 1738), 18.v.1972 (1 ♂, 1 ♀ with genitalia on slide BL 1711) (E.G., I. and E.A. Munroe) (CNC). FRENCH GUIANA: 5 ♂, 1 ♀, 36km SE, Roura (Camp Patawa), 21.xi.2007 (1 ♂, genitalia on slide MHNG ENTO 6239), 29–30.xi.2007 (3 ♂, 1 ♀, one ♂ with wings on slide MHNG ENTO 6272, 2 ♂ used for DNA sequencing and barcoding, one with labels LEP 963, BC MTD 01703, genitalia on slide TL 1, one with labels LEP 964, BC MTD 01704, genitalia on slide TL 2, ♀ with genitalia on slide MHNG ENTO 6240), 30.xi.2007 (1 ♂) (MHNG); 1 ♀ (abdomen used for DNA sequencing LEP 1290, genitalia on slide BL 1750) with same data as holotype; 1 ♂, same data as holotype except 2.ix.2011 (Hermier n° 24755); 1 ♀, same data as holotype except /604/ and 4.iii.2011 (Hermier n° 24344); 2 ♂, Beauséjour, N[ationale] 1 pk 28.5, 4°42'30"N, 52°23'30"W, 3.vi.2011, piège lumineux (Hermier n° 24545 & 24546) (B. Hermier) (MHNG); 1 ♂, Route d’Apatou pk 25.5 spk 2+4.4, 1.x.2011, piège lumineux (B. Hermier) (Hermier n° 24956) (MHNG); 1 ♂ (genitalia on slide BL 1749), R[ou]te forestière de Saut Léodate pk 4.5, 4°55'N, 52°33'W, 31.x.1995 piège lumineux (B. Hermier) (Hermier n° 8457) (MHNG).

**Other specimens.** 1 ♀ (genitalia on slide GS-5949-SB), Nova Olinda, Rio Purus, v.1922 (S. M. Klages) (CMNH); 1 ♀ (genitalia on slide Pyralidae Brit. Mus. Slide N° 17693), Teffé [sic], vi.1906 (W. Hoffmanns) (BMNH).

COI barcode sequence of paratype BC MTD 01703 (654 bp): ACTTTATATTTTATCTTTGGAATTTGAGCAGGAATAATTGGAACATCCTTAAGACTACTAATTCGAGCAGAATTAGGTAATCCTGGATCTCTTATCGGGGATGACCAAATTTATAACACTATTGTTACTGCTCATGCATTTGTAATAATCTTTTTTATAGTTATACCAATTATAATTGGTGGATTTGGAAACTGATTAGTACCTTTAATGCTAGGGGCACCAGATATAGCATTCCCTCGTATAAATAATATAAGATTTTGACTTCTTCCCCCCTCTTTAACCCTATTAATTTCAAGTAGAATTGTAGAAAATGGGGCAGGAACAGGATGAACCGTTTATCCACCTTTATCATCTAATATTGCCCATGGAGGCAGATCAGTAGATCTGGCAATTTTTTCACTACATTTAGCTGGAATTTCATCAATTTTAGGGGCAATTAATTTTATTACAACAATTATTAATATACGAATTAATAATCTTTCATTTGATCAAATACCCCTATTTGTTTGATCAGTAGGTATTACAGCATTACTATTACTTCTATCTTTACCAGTATTGGCGGGAGCTATTACCATACTTCTAACTGACCGAAATCTCAATACTTCCTTTTTTGATCCAGCAGGGGGGGGAGACCCTATTTTATATCAACACCTA

#### Diagnosis.

From *Catharylla gigantea*, *Catharylla chelicerata* differs in having the male costal arm hook shaped, longer, and thinner than in *Catharylla gigantea*, and the juxta is strongly downcurved, apically conical whereas it is long, almost straight, without apical conical projection downward in *Catharylla gigantea*. In female genitalia the sterigma forms a strongly sclerotized symmetrical structure made of two asymmetrical bell-shaped cavities, opened anterad in *Catharylla chelicerata* whereas it forms a pair of shallow pockets opened posterad in *Catharylla gigantea*.

#### Description.

Male (n = 21) (Figs 2, 9): Head with ochreous to brown chaetosemata. Antenna greyish brown with light brown scales, with patch of brown scales at base. Maxillary palpus ochreous to dark brown, lightly ringed with dark brown at 2/3, white tipped. Labial palpus: 1.3–2.0 mm long; ochreous, basally white, tip of segment II light greyish-brown; white tipped. Thorax with dark brown patch at collar. Foreleg coxa white; femur white, ashen brown dorsally, tibia and tarsomeres ochreous, distally ringed with dark brown. Midleg femur white, tibia ashen brown basally, tarsomeres ochreous, brown to ashen brown on upperside, with white ringed tips. Hindleg white, tarsomere I ochreous; II–V brown on upperside, with white ringed tips. Abdomen dull white to light ochreous. Forewing length: 10.5–15.0 mm; costal band wide, brown from base to apex; median and subterminal transverse lines faded brown, sometimes completely faded; dark brown spots on apical margin forming more or less continuous line; fringes brass colored; underside white with costal margin brown; outer margin with somewhat triangular spots. Hindwing snow white, with marginal spots between veins; fringes white; underside silvery white with marginal spots pronounced.

Tympanal organs (n = 9): Transverse ridge almost straight medially. Tympanic pockets conical, extending slightly beyond transverse ridge. Tympanic bridge lightly sclerotized, dorsal base of praecinctorium sclerotized. Tympanic drums elongate, bean shaped, posteriorly reaching transverse ridge or slightly beyond.

Male genitalia (n = 9) (Figs 13, 14): Uncus straight, of about 4/5 length of tegumen arms, dorso-ventrally compressed, with setae dorsally and laterally; apex truncated, slightly rounded, tip with short projection pointing posterad, ventrally convex, sometimes with median bump. Gnathos arms joining at 1/5 of length, regularly hook shaped, forming angle of about 100° with axis of basal arms, about 1/4 longer than uncus. Tegumen arms narrow at base, enlarging progressively toward dorsum to 2 × basal width, projected dorsally with bump at connection, connecting at distal 1/6. Cucculus densely setose, slightly directed upward on distal 1/3, apically truncated; basal 2/3 of costa of valva dorso-ventrally and laterally widened; costal arm hook-shaped, strongly sclerotized, directed upward at about 45° from costal arm base. Juxta triangular, curved downward, tip rounded and sac-like, basal lateral lobes curved ventrally. Saccus short, curved upward medially. Phallus narrow, S-shaped; vesica covered with tiny spicules, with one large, curved, pointed cornutus apically, preceded by string of 13–14 smaller cornuti increasing in size toward apex.

Female (n = 4): Labial palpi: 1.8–2.2 mm long. Forewing: 15–19.5 mm. Frenulum quadruple.

Female genitalia (n = 3) (Fig. 35): Papillae anales dorsally strongly produced posterad, with rounded bulge dorso-apically; ventrally slightly produced. Posterior apophyses 0.3–0.45 × length of papillae. Tergite VIII about half of length of sternite VIII. Anterior apophyses about 0.1 × length of papillae anales, slightly wider and rounded apically. Sternite VIII narrowing ventrally, densely covered with spinules, slightly connected medially at lamella antevaginalis; lamella antevaginalis slightly projected downward. Sterigma forming strongly sclerotized ventro-laterally symmetrical structure made of two asymmetrical bell-shaped cavities in ventral view, opened anterad, with dorsal lobe longer, expanding upward, slightly indented along latero-anterior margin; covered with minute punctuation. Ventro-basal section of ductus bursae tongue shaped, strongly sclerotized; ductus bursae long, ventrally sclerotized, widened and looped in basal half; enlarging progressively into corpus bursae. Corpus bursae egg-shaped with one signum.

#### Distribution.

The species was found in French Guiana and Brazil (Amazonas) (Fig. 43).

#### Etymology.

“*Chelicerata*” refers to the shape of the costal arms of the male valva, which look like mygalomorph chelicerae.

#### Notes.

Two females included here have been named *Catharylla robustella* (genitalia on slide GS-5949-SB, CMNH) and *Catharylla tenellina* (genitalia on slide Pyralidae Brit. Mus. Slide N°17693, BMNH) by S. Bleszynski, as indicated on labels, but these names were never published. These two specimens are probably *Catharylla chelicerata*, but the bad genitalia preparations do not allow to see details, and therefore they are not included as paratypes.

### Catharylla gigantea

T. Léger & B. Landry sp. n.

http://zoobank.org/06B7837B-A5DE-4347-824E-B74127F028E2

http://species-id.net/wiki/Catharylla_gigantea

Figs 3 , 15 , 16 , 36 , 43

#### Type material.

**Holotype.** ♂, with labels as follows: “Brazil: Amazonas, Manaus, | Reserva Ducke, AM-010, k[ilo]m[eter]. 26 | 2°55'S, 59°59'W, Dec[ember].13, 1993 | J. Bolling Sullivan & | Roger W. Hutchings | U[ltra]V[iolet] Light (Plateau Hut)”; “HOLOTYPE | Catharylla gigantea | T. Léger & B. Landry” [red label]; “BL 1747 ♂” [light green label]. Deposited in USNM.

**Paratypes.** 5♂, 2 ♀. BRAZIL: 1 ♂, Amazonas, Reserva Ducke, km. 26, Manaus–Itacoatiara Highway, 15.v.1972 (E. G., I. and E. A. Munroe) (CNC). FRENCH GUIANA: 1 ♂, 1 ♀ (genitalia respectively on Pyralidae Brit. Mus. slides N° 11224 and 11342), Saint-Jean-du-Maroni (E. Le Moult) (BMNH); 1 ♂ (genitalia on slide GS-6694-SB), Oyapok [sic] River, Pied Saut, iii.1918 (S. M. Klages) (CMNH). GUYANA: 2 ♂, 1 ♀ (1 ♂ with genitalia on slide BL 1716, ♀ with genitalia on Pyralidae Brit. Mus. Slide N° 19017), Potaro, ii.1908 (2 ♂), v.1908 (1 ♀) (S. M. Klages) (BMNH).

#### Diagnosis.

From *Catharylla chelicerata*, *Catharylla gigantea* differs in having the male costal arm shorter, basally wide and tooth shaped while it is long, narrow throughout and hook shaped in *Catharylla chelicerata*. The juxta is long, tongue shaped, almost straight, and apically rounded, whereas it is downcurved and apically conical in *Catharylla chelicerata*. In female genitalia, the sterigma forms a pair of shallow pockets opened posterad whereas in *Catharylla chelicerata* the sterigma forms a strongly sclerotized symmetrical structure made of two asymmetrical bell-shaped cavities opened anterad.

#### Description.

Male (n = 6) (Fig. 3): Head with ochreous chaetosemata. Antenna brown with light brown scales, with patch of dark brown scales at base. Maxillary palpus brown with dark brown spot at half of length, white tipped. Labial palpus: 1.6–2.4 mm long; ochreous to brown ochreous, basally white, with patch of dark brown scales at half of length, white tipped. Thorax with some brown at collar. Foreleg coxa white, femur white, ashen brown dorsally; tibia and tarsomeres brown-ochreous, distally ringed with dark brown. Midleg white with tibia-femur joint and base of tibia ashen; tarsomeres ochreous to brown ochreous with upperside brown to dark brown, white tipped. Hindleg white with tarsomeres II–V ochreous to brown ochreous, upperside brown, with white tips. Forewing length: 13.5–14.5 mm; snow white with wide brown to dark brown costal line from base to apex; median and subterminal transverse lines faded brown; dark brown spots on termen forming more or less continuous line; fringes brass colored; underside white, with costal margin brown ochreous, outer margin with subtriangular spots. Hindwing snow white; marginal spots dark brown between R5, M1, M2, M3, and CuA1; fringe white; underside snow white, with same spots as on upperside.

Tympanal organs (n = 5): Transverse ridge medially convex. Tympanic pockets extending slightly beyond transverse ridge, rounded. Tympanic bridge lightly sclerotized, dorsal base of praecinctorium sclerotized. Tympanic drums elongate, bean shaped.

Male genitalia (n = 5) (Figs 15, 16): Uncus straight, about 3/4 length of tegumen arms, dorso-ventrally flattened, dorsally convex, ventral margin convex in basal half, concave in distal half; basally and laterally setose; apex slightly rounded, medially with short projection pointing postero-ventrally. Gnathos arms joining at 1/5, about 1/4 longer than uncus, regularly curved. Tegumen arms narrow at base, widening regularly to reach 1.5 × basal width dorsally, with connection at distal 1/6. Cucculus densely setose, broad at base, slightly widening and truncate at apex; costal arm of valva basally wide, short, tooth shaped, slightly curved inward. Juxta long, tongue shaped, almost straight, apically rounded, with basal lateral lobes curved ventrally. Saccus short, curved upward. Phallus narrow, S-shaped; vesica covered with tiny spicules, with string of 14 small cornuti increasing in size toward apex, with apical cornutus up to 5 × length of previous one.

Female (n = 2): Labial palpi: 2.5–3.1 mm long. Forewing length: 17.5–22 mm; frenulum triple.

Female genitalia (n = 2) (Fig. 36): Papillae anales ventrally projected. Posterior apophyses about 0.35 × length of papillae anales, narrow. Segment VIII narrowing ventrally, densely covered with spinules; narrow connection at lamella antevaginalis; lamella antevaginalis slightly projected downward. Sterigma forming pair of shallow pockets opened posterad at base of segment VIII. Anterior apophyses about 0.08 × length of papillae anales, of medium width, basally wide. Ductus bursae wide, as long as twice segment VIII, regularly enlarging into corpus bursae. Corpus bursae with one rounded signum.

#### Distribution.

*Catharylla gigantea* has been found in French Guiana, Guyana, and Brazil (Amazonas) (Fig. 43).

#### Etymology.

The name comes from the Latin *giganteus, a, um* meaning very large.

#### Notes.

The name was given to the species on manuscript labels by S. Blezynski, probably in reference to the large size of the female.

### *Tenellus* species group

**Diagnosis.** The synapomorphies of the group are the dorsal furrow on the uncus, the uncus apex slightly bifid, the presence of a transtilla in male genitalia, and the absence of a ventral connection of sternite VIII in female genitalia. The *tenellus* species group can also be separated from the other *Catharylla* species based on the following additional diagnostic characters: the hindwings are creamy-white, and in female genitalia, the papillae anales are not produced.

**Notes.** This group includes three species, including two new ones. *Catharylla serrabonita* and *Catharylla tenellus* form a monophyletic group within the *tenellus* species group (Fig. 42). Relationships to other species groups are unknown.

### Catharylla tenellus

(Zeller, 1839)

http://species-id.net/wiki/Catharylla_tenellus

Figs 4 , 17 , 18 , 27–29 , 37 , 45

Crambus tenellus Zeller, 1839: 174–175
Catharylla tenella : Zeller 1863: 50; Bleszynski and Collins 1962: 226; Landry 1993: 1088; Munroe 1995: 35; Nuss et al. 2013.
Argyria tenella : Zeller 1877: 58; Dyar 1914: 317
Platytes tenella : Hampson 1896: 944

#### Type material.

**Holotype.** ♀, “Type” [red ringed]; “Catharylla | tenella Z[eller]. | Mon[ograph]. p[age]. 50 Am. anftr.” [not clearly readable]; “Zell[er]. Coll[ection]. | 1884”; “♀ | Pyralidae | Brit[ish]. Mus[eum]. | Slide N° | 1094”. Deposited in BMNH.

**Other specimens examined.** 20 ♂, 7 ♀. BRAZIL: 3 ♂ (1 ♂ with leg used for DNA barcoding BC MTD 01842, 1 ♂ with genitalia on slide BL 1757), São Paulo, Ubatuba, Picinguaba, 23°22'S, 44°50'W, 2–20m, 22–24.ix.2001 (V. O. Becker n°132820) (Becker Coll.); 2 ♂ with same data except 10–12.xi.2001 (V. O. Becker n°133712) (Becker Coll.); 2 ♂, 1 ♀ (1 ♂ with genitalia on slide BL 1741, ♀ genitalia on slide BL 1742), São Paulo, Bertioga, 5 m, 5.xi.1995 (V. O. Becker n°99090) (USNM); 1 ♂ (genitalia on slide BL 1778) with same data (Becker Coll.); 1 ♂ (genitalia on slide BL 1775) with same data except 15–17.v.1996 (V. O. Becker n° 99386) (Becker Coll.); 1 ♂ with same data except 7–9.x.1996 (V. O. Becker Coll. n°99757) (Becker Coll.); 1 ♂ (used for DNA sequencing and barcoding LEP 975, BC MTD 01711, genitalia on slide TL 13), São Paulo, São Luiz do Paraitinga, 23°20'S, 45°06'W, 900 m, 13–20.iii.2001 (V. O. Becker n°132356) (Becker Coll.); 1 ♂ (genitalia on slide Pyralidae Brit. Mus. Slide N° 19065), São Paulo, 700 m (E. D. Jones) (BMNH); 1 ♂ (genitalia on slide BL 1746), Minas Gerais, Caraça, 1300 m, 1–2.iv.1992 (V. O. Becker n°85081) (USNM); 1 ♀ (genitalia on slide BL 1754) with same data (Becker Coll.); 1 ♂ (genitalia on slide BL 1755) with same data except 25.x.1994 (V. O. Becker & K. S. Sattler, n°93291) (Becker Coll.); 1 ♂, 1 ♀ (♂ used for DNA sequencing and barcoding LEP 973, BC MTD 01709, genitalia on slide TL 11, ♀ genitalia on slide BL 1758), Bahia, Porto Seguro, A. d’Ajuda, 16°27'S, 39°03'W, 20 m, 12.vii.2009 (V. O. Becker n°144140) (Becker Coll.); 2 ♂ (used for DNA sequencing and barcoding, one with labels LEP 972, BC MTD 01888, genitalia on slide TL 10, other with labels LEP 974, BC MTD 01710, genitalia on slide TL 12) with same data except 15.viii.2008 (V. O. Becker n°140808) (Becker Coll.); 1 ♂ (used for DNA sequencing and barcoding LEP 971, BC MTD 01708, genitalia on slide TL 9) with same data except 1–3.v.2009 (V. O. Becker n°142784) (Becker Coll.); 1 ♂, Paranà, Castro (USNM); 1 ♀ (genitalia on slide BL 1733), Rio de Janeiro, xi[day and year data missing] (H. H. Smith) (CMNH); 1 ♀ (genitalia on Pyralidae Brit. Mus. Slide N°19069), Rio de Janeiro, Corcovado, 457 m, 26.xii.1958 (E. P. Wiltshire) (BMNH). No locality data: 1 ♂, 1 ♀ (♂ genitalia on slide Nat[ur]. hist[orisches]. Mus[eum]. Wien Gen[italia]. Praep[aration]. MV 9022a, ♀ genitalia on slide Nat. hist. Mus. Wien Gen. Praep. MV 9022b); 1 ♀ (genitalia on slide Nat. hist. Mus. Wien Gen. Praep. MV 9022c), 1869 (NMW).

COI barcode sequence of specimen BC MTD 1710 (654 bp): ACTCTATATTTTATCTTTGGAATTTGATCAGGAATAATTGGAACATCTTTAAGATTATTAATTCGAGCAGAATTAGGGAATCCTGGATCTCTAATTGGAGATGATCAAATTTATAACACTATTGTAACAGCCCATGCATTTATTATAATTTTTTTTATGGTTATACCAATTATAATTGGTGGATTTGGAAATTGATTGGTTCCATTAATATTAGGAGCCCCAGATATAGCTTTCCCCCGAATAAATAACATAAGATTTTGGTTATTACCCCCTTCCTTAACTCTTTTAATTTCTAGAAGAATTGTAGAAAATGGAGCTGGAACAGGATGAACGGTCTACCCCCCCCTTTCATCTAATATTGCCCATAGTGGAAGATCTGTAGATTTAGCAATCTTTTCTCTTCATTTAGCTGGAATTTCATCAATTTTAGGAGCTATTAATTTTATTACAACAATTATTAATATACGAATTAGTAATTTATCTTTTGATCAAATACCTTTATTTGTTTGATCAGTCGGTATTACAGCTTTACTTCTTCTTCTATCTTTACCTGTATTAGCAGGAGCTATTACTATACTTTTAACTGATCGAAATTTAAATACATCTTTTTTTGATCCTGCAGGAGGAGGAGATCCTATCTTATATCAACATTTA

#### Diagnosis.

From *Catharylla serrabonita* and *Catharylla coronata*, *Catharylla tenellus* can be separated by the median transverse line, which is faintly convex towards costa, whereas it is more strongly convex in *Catharylla coronata* and *Catharylla serrabonita*. The male genitalia provide the best diagnostic characters. The most obvious refers to the transtilla, which forms a pair of short, narrow sclerotized arms with pointed tips, projecting posterad, with, in between, a pair of brushes directed medio-ventrally, whereas it forms a pair of arms pointing posterad with a string of spines ventrally in *Catharylla serrabonita* and *Catharylla coronata*. In female genitalia, the anterior angle of sternite VIII is directed downward into a more or less rounded projection covered with short spinules of same length, whereas it is projected anterad in *Catharylla serrabonita*, and it is not projected in *Catharylla coronata*.

#### Redescription.

Male (n = 20) (Fig. 4): Head white with ochreous chaetosemata. Antenna brown with ochreous scales. Maxillary palpi ochreous to brown, white tipped. Labial palpi: 1.1–1.4 mm long; basally white, medially brown ochreous with white tips. Thorax slightly ochreous at collar. Foreleg coxa white; femur ochreous, dorsally dark brown; tibia and tarsomeres ochreous, distally ringed with dark brown. Midleg light ochreous with tibia-femur joint brown; tarsomeres II–V dark brown on upperside, with white ringed tips. Hindleg white; tarsomeres as midleg. Forewing length: 10.5–12 mm; snow white; costal line ochreous, lightly pronounced from base to apex; median and subterminal transverse lines ochreous, median transverse line faintly convex towards costa; outer margin ochreous with 7 dark brown spots often triangular, strongly pronounced; fringes brass colored; underside ochreous with costal margin pronounced in basal half and marginal spots pronounced. Hindwing cream-coloured; outer margin with small ochreous brown spots forming more or less continuous line between Sc+R1, Rs, M1, M2, M3, CuA1 and CuA2; underside light ochreous, with marginal spots pronounced; fringes white.

Tympanal organs (n = 13): Transverse ridge more or less regularly rounded, medially more straight. Tympanic pocket extending slightly beyond transverse ridge. Tympanic drum ovoid, posteriorly not extended beyond transverse ridge. Tympanic bridge faintly sclerotized.

Male genitalia (n = 13) (Figs 17, 18, 27–29): Uncus about half of length of tegumen arms, broadly downcurved; uncus arms connecting at base, with ventro-lateral tuft of setae; dorsal furrow with few short setae on each side, tip rounded, slightly indented medially, slightly convex in apical 1/3. Gnathos short and thick, arms joining at half of length, laterally compressed toward apex, almost straight, slightly downcurved, with apex pointing upward. Tegumen arms slightly enlarging toward apex; connecting at 3/4, slightly projected dorsally at connection. Costa of valva at 2/3 with arm directed postero-dorsally with rounded tip, without basal projection on dorsal edge or narrow with low basal projection, or wide with basal projection; cucculus upcurved in apical 1/4. Juxta ogival, posteriorly directed downward, with pair of thumb-like lobes ventrally reaching about 2/3 of length. Transtilla strongly sclerotized, with pair of narrow arms on each side of middle, pointing posterad, with 3 spines apically, also with pair of shorter brushes of tightly set spines medio-ventrally; in some specimens triangular median projection dorsally with few tiny setae. Phallus S-shaped, subapically with dorsal bump, apically lightly sclerotized, truncated, covered with microspicules barely visible; vesica without cornuti.

Female (n = 7): Labial palpi: 1.6–2 mm; forewing length 12–16 mm; frenulum triple.

Female genitalia (n = 7) (Fig. 37): Papillae anales straight, thick. Posterior apophyses narrow 0.3–0.45 × length of papillae anales, slightly wider basally. Intersegmental membrane between segments VIII and IX covered with microspines. Tergite VIII laterally about 2X longer than dorsally; sternite VIII formed by 2 lobes regularly narrowing downward in more or less triangular shape, not connected ventrally, densely covered with short spinules of same length; ventro-anterior angle of sternite VIII slightly projected downward, rounded, covered with short spinules; anterior margin of segment VIII latero-dorsally strongly sclerotized, thicker; posterior margin with dorsal line of setae. Anterior apophyses 0.02–0.05 × length of papillae anales. Sterigma membranous, covered with spinules. Ductus bursae about 3× length of corpus bursae, narrow. Corpus bursae elongate, sometimes with one signum.

#### Distribution.

The species is known from Brazil in the Atlantic Forest (Bahia, Minas Gerais, Paraná, Rio de Janeiro, Saõ Paulo) (Fig. 45).

Notes. The species was described from “one female collected in Brazil, near Rio de Janeiro”. Hence, the lectotype designated by a label by S. Bleszynski is not warranted. This designation is presumably based on the fact that Zeller (1863: 50) mentions a pair deposited in the Vienna Museum. The association of sexes in this species is not 100% certain.

Specimens from Porto Seguro, Brazil show a divergence of 3.34% in COI barcode sequences with the specimen from Ubatuba, Brazil. In morphology, differences in male genitalia are also observed: in the specimens from Bertioga, Caraça, São Paulo and Ubatuba the costal arm of the valva is wide and 1/3 of the length of the cucullus, almost reaching its tip, and the dorsal edge at base is slightly produced (Fig. 27). In the specimens from Porto Seguro, the costal arm is about 1/5 the length of the cucullus, relatively narrow, and the dorsal edge is slightly produced at base (Fig. 29). Another form, from Caraça, Minas Gerais (Fig. 28) was also found. No differences were found in the female genitalia. We feel that specimens and data are currently lacking to conclude that possibly more than one taxon should be recognized under *Catharylla tenellus*, or that there is indeed a deep divergence in the COI barcode between populations of this species.

### Catharylla coronata

T. Léger & B. Landry sp. n.

http://zoobank.org/7E0EB0BE-44C4-42EC-9F4D-2C923E9299E6

http://species-id.net/wiki/Catharylla_coronata

Figs 5 , 19 , 20 , 38 , 45

#### Type material.

**Holotype.** ♂, with labels as follows: “Col. BECKER | 81552”; “BRASIL:ES | Linhares, 40m | 20-29.ii.1992 | V.O.Becker Col”; “HOLOTYPE | Catharylla | coronata | Léger & Landry” [red label]. Deposited in Becker Collection.

**Paratypes.** 21 ♂, 4 ♀. BRAZIL: 5 ♂ with same data as holotype (1 used for DNA barcoding BC MTD 01890, 1 with genitalia on slide BL 1743); 2 ♂ with same data as holotype (1 used for DNA barcoding BC MTD 01891) except 05–09.iv.1992 (V. O. Becker n°82486); 6 ♂, 1 ♀ (1 ♂ with genitalia on slide BL 1730, ♀ with genitalia on slide BL 1731), Paranà, Rio Negro, 900 m, 8.ii.1973 (2 ♂), 10.ii.1973 (1 ♂), 11.ii.1973 (3 ♂), 13.ii.1973 (1 ♀) (A. & J. Razowski) (ISZP); 2 ♂, 2 ♀, Paranà, Curitiba, 920 m, 17.ii.1975 (1 ♂) (V. O. Becker n°10167), 20.ii.1975 (1 ♀, genitalia on slide BL 1756) (V. O. Becker n°10168), 12.iii.1975 (1 ♀, genitalia on slide BL 1753) (V. O. Becker n°10166), 10.x.1975 (1 ♂) (V. O. Becker n°4010) (Becker Coll.); 1 ♂ (genitalia on Pyralidae Brit. Mus. Slide No. 11357), Paranà, Castro, 950 m (E. D. Jones) (BMNH); 1 ♂, Paranà, Quatro Barras, 850 m, 27.ii.1970 (Laroca & Becker) (V. O. Becker n°15442) (Becker Coll.); 1 ♂ (genitalia on Pyralidae Brit. Mus. Slide No. 11337) Rio de Janeiro, Novo Friburgo (BMNH); 1 ♂ (genitalia on Pyralidae Brit. Mus. Slide. No. 19019) Sao Paulo, 700 m (E. D. Jones) (BMNH); 1 ♂, 1 ♀ (♂ with genitalia on slide BL 1774, ♀ with genitalia on slide BL 1736), Santa Catarina, Rio Vermelho, 968 m, 18.ii.1973 (♂), 28. ii. 1973 (♀) (A. & J. Razowski) (ISZP); 1 ♂, no locality data (V. O. Becker) (Becker Coll.).

COI barcode sequence of paratype BC MTD 01890 (654 bp): ACTTTATATTTTATTTTTGGAATTTGAGCAGGAATAGTAGGAACATCATTAAGATTATTAATTCGAGCTGAATTAGGTAATCCTGGATCTCTTATTGGAGATGATCAAATCTATAATACTATTGTAACCGCTCATGCATTTATTATAATTTTTTTTATAGTTATACCAATTATAATTGGTGGATTTGGAAATTGATTAGTTCCCTTAATATTAGGAGCACCAGATATAGCTTTTCCTCGAATAAATAACATAAGATTTTGATTATTACCCCCCTCTTTAACTCTTTTAATTTCAAGAAGAATTGTAGAAAATGGAGCTGGAACAGGATGAACAGTTTACCCCCCACTTTCATCTAATATTGCCCATAGTGGAAGATCCGTAGATTTAGCAATCTTTTCCCTTCATTTAGCTGGAATTTCTTCAATTTTAGGAGCAATTAATTTTATTACAACAATTATTAATATACGAATCAATAATCTTTCATTTGATCAAATACCTCTTTTTGTTTGATCAGTAGGAATTACAGCTTTACTTCTTCTTTTATCATTACCAGTATTAGCTGGAGCTATTACTATACTTTTAACTGATCGAAATTTAAATACATCTTTTTTTGATCCCGCAGGAGGAGGAGATCCTATTTTATATCAACATTTA

#### Diagnosis.

From *Catharylla serrabonita* and *Catharylla tenellus*, *Catharylla coronata* can be separated with characters of the male genitalia: the uncus is apically bifid and grooved on distal 1/5 in *Catharylla coronata* whereas it is only indented medially at apex in *Catharylla serrabonita* and *Catharylla tenellus*; the costal arm of the valva is short and the apex is curved inward in *Catharylla coronata* whereas the costal arm is longer and points postero-dorsally in the other two species; the transtilla forms a pair of sclerotized arms slightly bent inward distally, ventrally with a row of short spines increasing in size from base to apex whereas it forms a pair of short, narrow sclerotized arms with pointed tips, projecting posterad, and with a pair of brushes directed medio-ventrally in *Catharylla tenellus* and a pair of sclerotized arms strongly bent inward on distal 1/4 and with a string of long spines of same length medially along it in *Catharylla serrabonita*; the juxta is shorter than in *Catharylla tenellus*, and regularly narrowing toward apex whereas it is strongly narrowing on distal 1/4 in *Catharylla serrabonita*; the ventral projections of the juxta form a pair of shallow pockets whereas they are bell-shaped in *Catharylla serrabonita* and thumb-like in *Catharylla tenellus*; the vesica has a row of 6–7 cornuti in *Catharylla coronata* whereas it does not show any cornuti in *Catharylla serrabonita* and *Catharylla tenellus*. In the female genitalia of *Catharylla coronata*, the anterior angle of sternite VIII is not projected whereas it is rounded, projected anterad and covered with short spinules in *Catharylla serrabonita*, and projected downward in *Catharylla tenellus*. The anterior apophyses are quadrangular, anvil shaped whereas they are spine like in the other two species.

#### Description.

Male (n = 21) (Fig. 5): Head white with ochreous chaetosemata. Antenna brown, with whitish ochreous scales and patch of brown scales at base. Maxillary palpi light ochreous to ochreous, white tipped. Labial palpi: 1.6–1.85 mm long; light ochreous, white tipped. Thorax white, with ochreous patch at collar. Foreleg coxa white; femur white, dorsally dark brown; tibia and tarsomeres ochreous, distally ringed with brown; midleg and hindleg white to light ochreous, tarsomeres II–V ochreous, upperside brown, with white ringed tips. Forewing length: 10–13 mm; costal margin line thin, light ochreous, apically faded; median transverse line light ochreous, concave on costal half, more or less disrupted; subterminal transverse line ochreous, curving toward base on costal half; R5 vein faintly marked apically with ochreous; outer margin ochreous with 7 pronounced dark brown spots more or less triangular between veins, sometimes connecting; fringes brass colored; underside white ochreous to ochreous, costal margin basally brown; outer margin with pronounced spots. Hindwing white to creamy white, usually with marginal brown spots between Sc+R1, Rs, M1, M2, M3, CuA1 and CuA2, forming more or less continuous line; fringes white; underside light ochreous, with dark brown marginal spots pronounced.

Tympanal organs (n = 7): Transverse ridge more or less regularly rounded. Tympanic pocket extending faintly beyond transverse ridge, rounded. Tympanic drum glomerular, not reaching transverse ridge.

Male genitalia (n = 7) (Figs 19, 20): Uncus about 3/4 length of tegumen arms, downcurved; uncus arms basally with ventro-lateral tuft of setae; dorsal furrow pronounced medially with row of few setae on each side; thin, bifid on distal 1/5, slightly grooved, with apex slightly pointed; with shallow cavity ventro-apically. Gnathos arms connecting at 1/3 of length; shaft slightly downcurved, with apex pointing upward. Tegumen arms enlarging progressively toward uncus; tegumen connection about 1/3 arms length. Costa of valva basally narrow, with quadrangular projection, apically narrowing into arm pointing posterad with short tip curved inward; cucullus curved upward in distal 1/3, with apex rounded. Juxta triangular, regularly narrowing toward apex with shallow pockets projected ventro-laterally; with baso-lateral angles curved upward. Transtilla modified into two arms projecting posterad, slightly curved inward in distal 1/4, with longitudinal string of short spines ventrally at base, medially along arms, and at apex, increasing in size from base to apex in factor of about 1 to 4–5. Phallus almost straight, apex dorsally triangular; vesica basally covered with tiny spicules, microspicules barely visible all along vesica, also with row of 5–6 straight, short spine-like cornuti wider at their base.

Female (n = 4): Labial palpi: 1.6–2.2 mm long. Forewing length 14–16 mm. Frenulum triple.

Female genitalia (n = 4) (Fig. 38): Papillae anales straight, thick. Posterior apophyses 0.3–0.5 × length of papillae anales, wide at base, about half of length of papillae. Intersegmental membrane between segment VIII and IX covered with microspines. Sternite VIII laterally about 1/3 longer than tergite VIII. Sternite VIII formed by 2 lobes regularly narrowing downward into triangle, not connected ventrally, densely covered with spinules, with spinules longer ventrally. Anterior apophyses about 0.05 × length of papillae anales, quadrangular, anvil shaped. Anterior margin of sternite VIII latero-dorsally strongly sclerotized, thicker; posterior margin with dorsal line of setae. Sterigma membranous, covered with spinules. Ductus bursae regularly enlarging into corpus bursae, basally directed downward. Corpus bursae more or less rounded, faintly delimited from ductus bursae, with one oval signum.

#### Distribution.

The species occurs in Brazil in the following states: Bahia, Espirito Santo, Paranà, Rio de Janeiro, Santa Catarina, Saõ Paulo (Fig. 45).

#### Etymology.

The name comes from the latin *coronatus, a, um*: crowned, referring to the longitudinal string of short spines of the transtilla in the male genitalia.

#### Notes.

Based on our combined phylogenetic analysis, *Catharylla coronata* is the sister species of the *Catharylla tenellus* + *Catharylla serrabonita* pair (Fig. 42).

### Catharylla serrabonita

T. Léger & B. Landry sp. n.

http://zoobank.org/8B0F3E46-1CA6-47C3-A5AC-D9A9A01AAA29

http://species-id.net/wiki/Catharylla_serrabonita

Figs 6 , 21 –26 , 39 , 45 , 46

#### Type material.

**Holotype.** ♂, with labels as follows: “BRASIL: BA, Camacan | Res[erva]. Serra Bonita | 15°23'S, - 39°33'W, | 800m, 06.iv.2011 | B. Landry, V. Becker”; “HOLOTYPE | Catharylla serrabonita | T. Léger & B. Landry” [red label]. Deposited in Becker Collection.

**Paratypes.** 21 ♂, 1 ♀. BRAZIL: 5 ♂ (1 used for DNA barcoding BC MTD 01843, 1 with genitalia on slide BL 1745), Espírito Santo, Linhares, 40m, 25–30.i.1998 (V. O. Becker n°113929) (Becker Coll., USNM); 2 ♂, 1 ♀ (♀ with genitalia on slide BL 1759) with same except 20–29.ii.1992 (V. O. Becker n°81552) (Becker Coll., USNM); 2 ♂ with same data as holotype except 05–09.iv.1992 (V. O. Becker n°82486) (USNM); 10 ♂ (1 in alcohol, thorax used for DNA sequencing LEP 979, genitalia on slide TL 7, wing on slide TL 8) Bahia, Camacan, Serra Bonita Reserve, 15°23'S, 39°33'W, 800 m, B. Landry, V. O. Becker, 1.iv.2011 (1 ♂), 2.iv.2011 (2 ♂), 3.iv.2011 (1 ♂, genitalia on slide BL 1776), 5.iv.2011 (3 ♂), 6.iv.2011 (1 ♂), 7.iv.2011 (1 ♂) (MHNG); 1 ♂ with same data except vii.2010 (V. O. Becker) (Becker Coll.); 1 ♂ (used for DNA sequencing and barcoding LEP 970, BC MTD 01887, genitalia on slide TL 6) Bahia, Porto Seguro, A. d’Ajuda, 16°27'S, 39°03'W, 20 m, 12.vii.2009 (V. O. Becker n°144140) (Becker Coll.).

COI barcode sequence of holotype LEP 979 (516 bp): TAGTTGGAACATCATTAAGACTATTAATTCGAGSAGAGTTAGGGAATCCTGGATCTCTTATTGGAGATGATCAAATTTATAATACTATTGKAACAGCTCATGSATTTATTATAATTTTTTTTATAGTTATACCAATTATAATTGGTGGATTTGGAAACTGACTAGTTCCATTAATATTAGGAGCCCCAGACATAGCTTTCCCCCGAATAAATAATATAAGATTTTGATTACTCCCCCCCTCTTTAACCCTTTTAATTTCCAGAAGAATTGTAGAGAATGGAGCTGGAACAGGATGAACGGTTTACCCCCCCCTTTCATCTAATATTGCTCATAGKGGAAGATCTGTAGATTTAGCAATTTTTTCTCTTCATTTAGSAGGAATTTCATCAATTTTAGGAGCAATTAATTTTATTACAACAATTATTAATATACGAATTAATAATTTATCTTTTGATCAAATACCGTTATTTGTCTGATCAGTTGGTATTACAGCTTTACTCCTTCTTTTATCTTTAC

#### Diagnosis.

From *Catharylla coronata* and *Catharylla tenellus*, *Catharylla serrabonita* can be separated by the zigzagging median transverse line with the short triangular dent at CuA2 and the pronounced creamy color of the hindwing. The male genitalia provide the best discriminant characters: in *Catharylla serrabonita*, the transtilla forms a pair of sclerotized arms bent inward in distal 1/4 and with a string of long spines of same length medially along it, whereas it forms a pair of short, narrow sclerotized arms with pointed tips projecting posterad, and with a pair of brushes directed medio-ventrally in *Catharylla tenellus*, and two sclerotized arms slightly bent inward distally, with a row of short spines increasing in size from base to apex in *Catharylla coronata*, and the juxta is apically narrow and pointed whereas it is triangular and regularly narrowed in *Catharylla coronata* and *Catharylla tenellus*. In female genitalia, the anterior angle of sternite VIII is projected anterad into a rounded protrusion covered with short spinules in *Catharylla serrabonita*, whereas it is projected downward in *Catharylla tenellus* and it is not projected in *Catharylla coronata*.

#### Description.

Male (n = 21) (Fig. 6): Head white with ochreous chaetosemata. Antenna brown with whitish-ochreous scales and patch of brown scales at base. Maxillary palpus light ochreous,with patches of dark brown scales at 1/3 and 2/3, white tipped. Labial palpus: 1.7–2.5 mm long; light ochreous, white tipped. Thorax white, with ochreous patch at collar. Foreleg coxa white; femur white, dorsally dark brown; tibia and tarsomeres ochreous, distally ringed with brown. Midleg and hindleg white to light ochreous; tarsomeres II–V ochreous, brown on upperside, with white ringed tips. Forewing length: 10–14 mm; costal line ochreous; median transverse line ochreous to brown, zigzagging with short brown pronounced spot at M1 and short triangular dent at CuA2; subterminal transverse line ochreous to brown, regularly curved up to CuA2, then curved again; R5 faintly marked apically with ochreous; outer margin ochreous with 7 more or less triangular and connected dark brown spots between veins; fringes brass colored; underside ochreous, outer margin with pronounced spots. Hindwing cream-coloured, usually with more or less connected marginal brown spots between Sc+R1, Rs, M1, M2, M3, CuA1 and CuA2; fringes white; underside light ochreous, with marginal spots pronounced.

Tympanal organs (n = 4): Transverse ridge more or less rounded, medially slightly flattened. Tympanic pocket extending faintly beyond transverse ridge, rounded. Tympanic drum glomerular, not reaching transverse ridge.

Male genitalia (n = 4) (Figs 21–26): Uncus about as long as tegumen arms, downcurved; uncus arms connecting basally, with ventro-lateral tuft of setae at base; dorsal furrow pronounced medially with row of few hairs on each side; apex rounded, slightly indented medially, slightly convex ventro-apically. Gnathos arms connecting at 1/3; main shaft slightly downcurved with apex pointing upward. Tegumen arms regularly enlarging toward apex, connection at about 4/5 length of arms. Costa with apically rounded arm pointing postero-dorsally; cucullus curved upward in distal 1/3, with apex rounded. Juxta triangular, narrowing in distal 1/4 with bell-shaped ventro-lateral projections, regularly curved with apex horizontally straightened; baso-lateral angles curved upward. Transtilla with two very large sclerotized arms projecting posterad, bent inward in apical 1/4, with longitudinal string of long spines medially. Phallus slightly S-shaped, with apex dorsally sclerotized; vesica covered with microspicules, without cornuti.

Female (n = 1): Labial palpi: 1.9 mm long. Forewing length: 14 mm. Frenulum triple.

Female genitalia (n = 1) (Fig. 39): Papillae anales straight, thick. Posterior apophyses 0.4 × length of papillae anales, narrow, wider at base. Intersegmental membrane between segment VIII and IX covered with microspines. Sternite VIII laterally about 5/3 length of tergite VIII; posterior margin of tergite VIII with line of setae; sternite VIII forming 2 triangular lobes regularly narrowing downward, not connected, densely covered with short spinules of same length; anterior angle of sternite VIII slightly projected anterad, rounded, covered with short spinules of same length. Anterior apophyses 0.03 × length of papillae anales. Sterigma membranous, covered with spinules. Ductus bursae about 3 × length of corpus bursae, narrow, basally directed downward and then bent upward. Corpus bursae elongate, ovoid, with one tiny signum.

#### Distribution.

The species occurs in Brazil (Bahia, Espirito Santo) (Figs 45 & 46).

#### Etymology.

The name comes from that of the Serra Bonita Reserve founded by Vitor O. Becker and Clemira de Souza. It is managed by Instituto Uiraçu in the State of Bahia, Brazil.

#### Notes.

Serra Bonita Reserve is located in the Atlantic Forest, in a hilly region of cacao plantations and scattered forest. Adults came late to light, usually after 23:00. Our molecular analysis of the COI barcode sequences highlighted that specimens from Serra Bonita respectively show 3.24 and 2.21 % base differences with those of Porto Seguro and Linhares. This divergence is possibly associated with slight morphological differences in male genitalia as shown in Figs 23–26. No females were found at Serra Bonita.

### *Mayrabonillae* species group

**Diagnosis.** We have not recovered any obvious synapomorphy for this group. It can be separated from the other *Catharylla* species based on the shorter forewing length, usually between 7.5 and 9.0 mm (maximum 10.5 mm). In male genitalia, the tegumen connection is more than two times longer than the uncus, the uncus is beak-shaped, with the apex narrowing to a point, the gnathos is bent at an angle of about 90°. In female genitalia, the basal line along the anal papillae is ventrally expanded onto a triangle, and the sterigma forms a pair of sclerotized pockets on each side of the middle, covered with short spines or spicules. The sterigma does not bear tiny setae on the ventral membrane of segment VIII.

**Notes.** This group includes two species. The phylogenetic analyses restricted to the nuclear genes and the combined Bayesian analysis place the *mayrabonillae* group as sister to the *chelicerata* group (Fig. 42).

### Catharylla mayrabonillae

T. Léger & B. Landry sp. n.

http://zoobank.org/5078E6D0-DDA4-4B9F-8089-F7FFECC725C6

http://species-id.net/wiki/Catharylla_mayrabonillae

Figs 7 , 30 , 31 , 40 , 44

#### Type material.

**Holotype.** ♂, with labels as follows: “Col. BECKER | 101668”; “ECUADOR: NAPO | Misahualli | 450m xii.1992 | V.O.Becker Col”; “HOLOTYPE | Catharylla | mayrabonillae | Léger & Landry” [red label]. Deposited in Becker Collection.

**Paratypes.** 16 ♂, 37 ♀. BRAZIL: 1 ♀ (genitalia on slide BL 1729), [Acre] Rio Branco, 1924 (Dengler) (SMNS); 2 ♀, Amazonas, Manaus, Reserva Ducke, AM-010, km 26, 2°55'S, 59°59'W, 15.xii.1993, U[ltra]V[iolet] Light (J. B. Sullivan & R. W. Hutchings) (USNM); 1 ♂ (genitalia on Pyralidae Brit. Mus. Slide No. 11341), Amazonas, Fonte Boa, ix.1906 (S. M. Klages) (BMNH); 1 ♀ (genitalia on slide BL 1713), Federal District, Estaçao Florestal, Cabeca do Vedao, 1100m, 18.x.1971 (E.G., I. & E.A. Munroe) (CNC); 2 ♂, Maranhão, Feira Nova, Faz[enda]. Retiro, 480m, 07°00'S, 46°26'W, 1–3.xii.2011 (V. O. Becker n°148263) (Becker Coll.); 2 ♀, Pará, Belém, 20m, i.1984 (V. O. Becker n°46981) (Becker Coll.); 1 ♀, Pará, Capitao Poco, 25–31.i.1984 (V. O. Becker n°97880) (Becker Coll.); 1 ♂, Rondonia, Cacaulãndia, 140m, xi.1991 (V. O. Becker n°79592) (Becker Coll.); 1 ♀, Rondonia, 62km S[outh] Ariquemes, Fazenda Rancho Grande, 165m, 10°32'S, 62°48'W, 18–26.iv.1991 (R. Leuschner) (USNM). COLOMBIA: 1 ♂, Valle, J[un]ct[ion]. Old B’[uena]v[en]tura R[oa]d. and Rio Dagua, 50m, 8.ii.1989 (J. B. Sullivan) (USNM). COSTA RICA: 1 ♀ (used for DNA Barcoding by Janzen, 07-SRNP-113921), Alajuela, Area de Conservacion Guanacaste, Estacion Caribe, 12.xi.2007 (S. Rios & H. Cambronero) (INBio); 1 ♀, Alajuela, Area de Conservacion Guanacaste, Rio Negro, 25.i.2009 (H. Cambronero & F. Quesada) (INBio); 2 ♂ (one used for DNA sequencing LEP 966, with genitalia on slide TL 3, other used for DNA sequencing LEP 967, with genitalia on slide TL 4), Alajuela, San Carlos, Arenal National Park, Send[ero] Pilón, Rio Celeste, 700m, light trap, 17–19.x.2001 (G. Rodriguez) (INBio); 1 ♂, Prov[incia] Guanacaste, F[in]ca Pasmompa, Est[acion] Pitilla, 5km SO S[an]ta Cecilia, 400m, xii.1990 (P. Rios & C. Moraga) (INBio). ECUADOR: 4 ♀ with same data and deposition as holotype; 4 ♂, 8 ♀ (1 ♂ used for DNA barcoding BC MTD 01844) with same data (USNM); 1 ♀ (genitalia on slide BL 1726, used for DNA sequencing and barcoding LEP 969, BC MTD 1707), Napo, 6km NW Tena, Lumu Caspi, 0°54'37"S, 77°49'32"W, 590m, 29.ix.2002 (Schouten Coll.); 1 ♀ (genitalia on slide BL 1715, used for DNA sequencing and barcoding LEP 968, BC MTD 1706), Pastaza, 1km N Santa Clara, 1°16'02"S, 77°52'57"W, 630m, 28.ix.2002 (Schouten Coll.). FRENCH GUIANA: 1 ♂, 4 ♀ (♂ genitalia on Pyralidae Brit. Mus. Slide No 7816, ♀ genitalia on slides BL 1720, BL 1725, and Pyralidae Brit. Mus. Slides No. 5953 and 19020), Saint Jean de Maroni (E. Le Moult) (BMNH); 2 ♀, Piste Nancibo, km 6, 4°41'N,; 52°25'W, in logged rain forest, at 125W[atts] mer[cury]-vapor light and 15W[atts] U[ltra]V[iolet], 11.i.1985 (J.[-]F. Landry) (USNM); 1 ♂, Roura, Montagne des Chevaux, xii.2008 (S. Delmas) (MHNG); 1 ♂ (genitalia on slide Pyralidae Brit. Mus. Slide No 7792), Cayen[ne] (BMNH). GUYANA: 1 ♀ (genitalia on slide BL 1723), Omai, vi.1908 (S. M. Klages) (BMNH); 1 ♀ (genitalia on slide BL 1722), Potaro i.1908 (S. M. Klages) (BMNH). PANAMA: 1 ♀, Rio Trinidad, 12.iii [no year data] (A. Busck) (USNM). PERU: 1 ♂ (genitalia on slide BL 1724), Agnaytia, Huallaga, 400m, ix.1961 (F. H. Walz) (CNC); 1 ♀ (genitalia on slide GS-6908-SB), Yurimaguas, Huallaga 14.iv.[19]20 (CMNH). SURINAME: 2 ♀ (genitalia on slides BL 1727 and BL 1728), Kabo, 5°16'N, 55°44'W, Saramaca, black light, respectively 15–16.iii.1983 and 13–14.i.1983 (K.E.Neerling) (Schouten Coll.); 1 ♀ (genitalia on slide BL 1710), Sipaliwini Distr[ict]., Tibiti area, Kabo Creek, partly swampy primary forest on hilly slopes, ca 2km from river, vi.1989 (J. Beerlink) (Schouten Coll.).

**Other specimen examined.** 1♀ (used for DNA sequencing Lep 1126), Peru, Huánuco, Rio Llullapichis, Panguana, 74,945°W / 9,614°S, 23.9.–10.10.2011 (SMTD).

COI barcode sequence of paratype 07-SRNP-113921 (654 bp): ACATTATATTTTATTTTCGGGATTTGAGCAGGTATAGTAGGAACTTCACTTAGATTATTAATTCGTGCTGAATTAGGTAACCCTGGCTCTCTTATTGGAGATGATCAAATTTATAATACTATTGTAACAGCCCATGCATTTATTATAATTTTTTTTATAGTTATACCTATTATAATCGGTGGATTTGGAAATTGATTAGTTCCTTTAATATTAGGGGCACCAGATATAGCTTTCCCTCGAATAAATAACATAAGATTTTGATTATTACCACCATCATTAACTCTTTTAATTTCTAGAAGAATTGTAGAAAATGGAGCTGGAACAGGATGAACAGTTTATCCACCTTTATCATCTAATATTGCCCATGGGGGTAGATCTGTAGATTTAACAATTTTTTCATTACATTTAGCTGGAATTTCATCAATTTTAGGAGCTATTAATTTTATTACAACAATTATTAATATACGAATTAATAATTTATCATTTGATCAATTATCATTATTTATTTGATCAGTAGGAATTACTGCTTTACTTTTATTATTATCATTACCAGTTTTAGCTGGGGCTATTACTATACTTTTAACTGATCGAAATCTTAATACATCATTTTTTGATCCAGCAGGAGGAGGAGATCCAATTTTATATCAACATTTA

#### Diagnosis.

The best discriminant characters externally between the two species of the *mayrabonillae* group are the shape of the forewing outer margin, which is slightly produced apically in *Catharylla mayrabonillae* and not produced in *Catharylla paulella*, and the forewing median transverse line with two strongly pronounced spots at 1/3 and 2/3 in *Catharylla paulella*, whereas these spots are lacking in *Catharylla mayrabonillae*. The hindwing of *Catharylla mayrabonillae* has a faded subterminal transverse line on costal half whereas the hindwing of *Catharylla paulella* lacks this marking. In male genitalia, the heavily sclerotized sacculus bears a dorso-lateral sclerotized string of short spines on distal 1/4 whereas the two processes of the costa are S-shaped in *Catharylla paulella*, and the apex of the phallus is trifid, rounded medially, shortly triangular laterally, whereas it is simply rounded in *Catharylla paulella*. In female genitalia, the sterigma forms double rounded cavities with a mustachio-shape arrangement of short spines in ventral view, and the ductus bursae is wide, progressively widening toward corpus in *Catharylla mayrabonillae*, whereas the sterigma forms a pair of shallow rounded pockets on each side of middle and the ductus bursae is narrow, with the rounded corpus bursae clearly differentiated from it in *Catharylla paulella*.

#### Description.

Male (n = 17) (Fig. 7): Head with light ochreous chaetosemata. Antenna brown, with white scales dorsally and patch of dark brown scales at base. Maxillary palpus light ochreous, ringed with dark brown at 2/3; white tipped. Labial palpus: 1–1.4 mm; white, with patch of dark brown scales at 1/3 and 2/3 laterally. Thorax with patch of light ochreous scales at collar. Foreleg coxa whitish brown, femur white, dorsally ashen brown, tibia and tarsomeres ochreous, distally ringed with dark brown. Midleg femur white, tibia light ochreous, basally brown, tarsomeres II–V ochreous with tips ringed white. Hindleg white, except tarsomeres, as in midleg. Abdomen dull white. Forewing length: 7.5–8.5 mm; with apex slightly produced; costal line thin, ochreous or white in basal half, white in apical half; median transverse line ochreous, slightly undulated; subterminal transverse line ochreous; transverse lines enlarging into brown spot on costal margin with ochreous bar on costa following subterminal transverse line; terminal sector with light ochreous between veins, margin with thin, dark brown line from apex to CuA1, with two dark brown spots in cubital sector, with spot between CuA1 and CuA2 slightly displaced toward base; fringes brass colored; underside light ochreous with some brownish scales, with thin brown margin. Hindwing white with thin transverse subterminal line faded ochreous, in continuity with forewing median transverse line; outer margin line pronounced, dark brown; underside dull white with thin faded brown margin; fringes white.

Tympanal organs (*n*=7): Transverse ridge regularly rounded, medially slightly flattened. Tympanic pockets broadly rounded, extended widely beyond transverse ridge, connected medially at base of praecinctorium. Tympanic drum bean-shaped, elongated, extended beyond tympanic pockets.

Male genitalia (n = 7) (Figs 30, 31): Uncus thick and wide, about 2/5 length of tegumen arms, densely setose, with shortly projecting apex dorsally rounded. Gnathos reaching about 1/4 longer than uncus; arms wide, joining at 2/5 of length; distal 2/5 at angle of about 85°. Tegumen almost regularly narrow, joined in last 1/4. Cucculus narrow, shorter than sacculus, apically rounded; sacculus greatly enlarged, thickly sclerotized, directed upward, then apically straight, slightly narrowing toward apex, laterally with string of short spines and 2–3 longer basal spines pointing downward; costal arm of valva directed upward, located at about 1/3 of costal margin, thin, strongly sclerotized, slightly curved. Vinculum arms narrow; saccus short and wide, tongue shaped, projecting posterad apically. Juxta elongate, distal 1/4 narrowed with rounded tip; wide base with ear-like lobes laterally and baso-lateral angle projected anterad. Phallus slightly bent sideways in distal 1/4, with trifid sclerotized apex rounded medially and shortly triangular laterally; vesica basally covered with tiny spicules, microspicules barely visible all along, with long spine-like, down-curved cornutus of about 2/5 length of phallus.

Female (n = 37): Labial palpi length: 1.1–1.3 mm. Forewing length: 9.5–10.5 mm; frenulum triple.

Female genitalia (n = 16) (Fig. 40): Papillae anales strongly curved in lateral view; sclerotized line along papillae expanding ventrally into triangle. Posterior apophyses 0.35–0.45 × length of papillae anales. Tergite VIII about 1/3 length of sternite VIII; postero-dorsal margin with few setae of moderate length; anterior apophyses 0.03–0.1 × papillae anales; sternite VIII with patches of minute setae antero-ventrally on each side of bare median band. Sterigma forming double rounded cavities with mustachio-shaped arrangement of short spines (in ventral view); remaining cavity wall with tiny spines. Ductus bursae short and wide, enlarged near middle; partly sclerotized on right side of enlargement and posterior section. Corpus bursae circular to elongate, about as long as tergite VII; single signum faintly pronounced.

#### Distribution.

The species has been found so far in Panama, Costa Rica, Colombia, Venezuela, Guyana, Suriname, French Guiana, Ecuador, Peru and Brazil (Acre, Amazonas, Distritò Federal, Pará, Rondônia) (Fig. 44). It is the most widespread species of *Catharylla* and the only one so far found in Central America and in Venezuela, Columbia, Ecuador and Peru.

#### Etymology.

*Catharylla mayrabonillae* is named in honor of Ms. Mayra Bonilla of San Jose, Costa Rica, in recognition of her artistic portrayal of the biodiversity and ecosystems of Costa Rica and her many years of support for the existence of the rain forest in Area de Conservacion Guanacaste.

#### Notes.

The relatively strong COI barcode divergence of 4.34% between samples LEP 1126 from Peru and 07-SRNP-113921 from Costa Rica (Table 5) is notable but it is not associated with morphological variation.

### Catharylla paulella

Schaus, 1922

http://species-id.net/wiki/Catharylla_paulella

Figs 8 , 10 , 32 , 33 , 41 , 44

Catharylla paulella Schaus, 1922: 131; Bleszynski and Collins 1962: 226; Bleszynski 1967: 97; Munroe 1995: 35; Nuss et al. 2013.

#### Type material.

**Holotype.** ♀, with labels as follows: “Sao Paulo | S.E. Brazil.”; “Collection | W[illia]mSchaus”; “Type No. | 25533 | U.S.N.M.” [orange label]; “SLIDE | SB ♀ | No.4641” [light blue label]; “Catharylla | paulella | type Sch[au]s” [hand written]; “Genitalia Slide | By SB | USNM 111,535” [green rectangular label with thin black line submarginally]. Deposited in USNM.

**Other specimens examined.** 2 ♂, 7 ♀. BOLIVIA: 2 ♂ (genitalia on slides GS-6652-SB and Pyralidae Brit. Mus. Slide No. 15890), Prov.[incia] del Sara, 450 m, iv.1910 (J. Steinbach) (CMNH, BMNH). BRAZIL: 1 ♀ (genitalia on slide BL 1752), Federal District, Planaltina, 15°35'S, 47°42'W, 1000 m, 3.xi.1977 (V. O. Becker n°22055) (Becker Coll.), 1 ♀ (genitalia on slide BL 1751) with same locality, 16.x.1990 (V. O. Becker n°96854) (Becker Coll.); 1 ♀, Maranhão, Feira Nova, Faz[enda]. Retiro, 480m, 07°00'S, 46°26'W, 1–3.xii.2011 (V. O. Becker n°148263) (Becker Coll.); 1 ♀ (genitalia on slide BL 1712), Mato Grosso, Urucum, 15 miles S[outh]. of Columbá, 650 f[ee]t, 19. iv. [19]27, at light (C. L. Collenette) (BMNH); 1 ♀, Parà, Belém, 20m, i.1984 (V. O. Becker n°46993) (Becker Coll.); 1 ♀ (genitalia on Pyralidae Brit. Mus. Slide N° 17692), São Paulo (BMNH); 1 ♀ (used for DNA sequencing and barcoding LEP 965, BC MTD 1705, genitalia on slide TL 5), São Paulo, São Luiz do Paraitinga, 23°20'S, 45°06'W, 900 m, 13–20.iii.2001 (V. O. Becker n°132357) (Becker Coll.).

COI barcode sequence of specimen LEP 965 (654 bp): ACATTATATTTTATTTTTGGAATTTGAGCAGGTATACTAGGAACTTCACTTAGATTATTAATTCGTGCTGAATTAGGTAATCCTGGATCTCTTATTGGTGATGATCAAATTTATAATACTATTGTAACAGCTCATGCATTTATTATAATTTTTTTTATAGTTATACCTATTATAATTGGTGGATTTGGAAATTGATTAGTTCCTTTAATATTAGGTGCACCAGATATAGCTTTCCCTCGAATAAATAATATGAGATTTTGATTATTACCCCCATCATTAACTCTTTTATTTT?TAGAAGAATTGTCGAAAATGGAACTGGAACAGGATGAACAGTTTACCCACCCTTATCATCCAATATTGCTCATAGAGGTAGATCAGTAGATCTAGCAATTTTTTCTTTACATTTGGCTGGAATTTCATCAATCTTAGGAGCTATTAATTTTATTACAACAATTATCAATATACGAATTAATAATTTATCTTTTGATCAATTATCATTATTTATTTGATCTGTAGGTATTACAGCTTTACTTTTATTATTATCATTACCAGTTCTAGCTGGAGCTATTACTATACTTTTAACTGATCGAAATCTTAATACATCATTTTTTGATCCTGCAGGAGGAGGTGATCCTATCTTGTATCAACATTTA

#### Diagnosis.

This species can be easily separated from the other *Catharylla* species by the forewing median transverse line with two strongly pronounced spots at 1/3 and 2/3. The forewing is also sparkled with dark brown scales, which is unique in the genus. In male genitalia, the two S-like projections of the costal arm of the valva discriminate this species from the other species of *Catharylla*. In female genitalia, the sterigma forms a pair of shallow rounded pockets on each side of middle, and the ductus bursae is narrow, with the rounded corpus bursae clearly differentiated from it in *Catharylla paulella*, whereas it forms double rounded cavities with a mustachio-shape arrangement of short spines in ventral view, and the ductus bursae is wide, progressively widening toward corpus in *Catharylla mayrabonillae*.

#### Redescription.

Male (n = 2): Head with ochreous chaetosemata. Antenna ochreous with white scales, with patch of dark brown scales at base. Maxillary palpus light ochreous, white tipped. Labial palpus: 1.4–1.7 mm long, light ochreous, white tipped. Thorax light ochreous at collar. Foreleg coxa whitish ochreous, femur light ochreous, dorsally ochreous; tibia and tarsomeres greyish brown, distally ringed with dark brown. Midleg and hindleg whitish ochreous; midleg tibia basally brown, hindleg tibia white; midleg and hindleg tarsomeres with white tips. Forewing length: 7–8 mm; costal line thin, brown or dirty white; median transverse line ochreous, with two dark brown strongly pronounced spots at 1/3 and 2/3; subterminal transverse line thin, ochreous, with small triangular spot on costal margin; with ochreous bar on costal margin following subterminal transverse line; outer margin ochreous with short dark brown lunules or dashes; fringes brass colored; underside light ochreous with brownish suffusion; with pronounced marginal spots. Hindwing white; outer margin with thin ochreous line in apical half; fringes white; underside dull white with dark brown marginal spots more or less connected on apical half.

Male genitalia (n = 2) (Figs 32, 33): Uncus almost straight, densely setose, about 1/4 length of tegumen arms. Gnathos with arms joining at 3/5, then directed upward at slightly less than 90° angle; apically narrowly rounded. Tegumen arms regularly widening toward uncus, connecting at about half their length. Cucculus of medium width, slightly curved upward in distal 1/4; costal arm of valva divided with short spatula at base and S-shaped projection with rounded apex apically. Juxta triangular with distal third narrower, apically rounded, with baso-lateral narrow, triangular projections pointing anterad. Phallus with apex more thickly sclerotized, with blunt apical margin, with short triangular ventral projection; vesica covered on basal 1/4 with tiny spicules, with barely visible microspicules all along, with one wide and curved cornutus at about 1/4 length of phallus.

Female (n = 7) (Figs 8, 10): Labial palpi: 1.4–1.7 mm long; forewing length: 9–9.5 mm; frenulum triple.

Tympanal organs (n = 5) (Fig. 10): Transverse ridge medially straight. Tympanic pockets not reaching beyond transverse ridge, rounded. Tympanic drum bean shaped, somewhat oval, just reaching transverse ridge.

Female genitalia (n = 5) (Fig. 41): Papillae anales ventrally slightly projected; sclerotized line at base enlarging medially to triangular shape covered by minute punctuation. Posterior apophyses 0.4–0.5 × length of papillae anales, narrow, tubular, with rounded tips. Tergite VIII short, about half of length of greatly enlarged sternite VIII. Anterior apophyses 0.05–0.1 × length of papillae anales. Lamella antevaginalis of sterigma dorsally covered with minute spicules; pair of shallow rounded pockets on each side of middle opened posterad. Base of ductus bursae sclerotized, forming circular membranous and narrow pocket. Corpus bursae regularly rounded, without signum.

#### Distribution.

The species has been found in Brazil (Federal District, Maranhão, Mato Grosso, Pará, Saõ Paulo) and in Bolivia (Fig. 44).

#### Notes.

The original description doesn’t mention the original number or sex of the specimens but it is assumed that there was only one. S. Bleszynski gave the new name of *Catharylla hibisca* to specimens that appear to be *Catharylla paulella*. The BMNH São Paulo specimen is associated with slide n° 17692, but the genitalia on this slide seem to be wrongly associated, given the inscription “wrong abdomen?” on the label, as well as the size of the abdomen, which is much bigger than those of *Catharylla paulella*. Therefore, this specimen cannot be identified with certainty. An error is possible in the association of the sexes of this species as there are no series of both sexes from the same locality or other means of associating them with 100% confidence.

### Key to the species of *Catharylla*

**Table:** 

1	Forewing costa with thick, brown to greyish brown stripe (Figs 2, 3); forewing length usually > 14 mm. Hindwing white. In male genitalia, gnathos regularly curved, juxta without latero-ventral projections (Figs 13, 15)	2
–	Forewing costa with thin, ochreous stripe or none (Figs 1, 4–8); forewing length 7–14 mm. Hindwing white, cream-coloured, or yellowish. In male genitalia, gnathos more or less straight, or bent at about 90° angle; juxta with latero-ventral projections (Figs 11, 17, 19, 21, 30, 32)	3
2	Male genitalia with costal arm long, narrow throughout, hook shaped, slightly curved, posteriorly reaching beyond cucculus (Fig. 13). In female genitalia, sterigma forming strongly sclerotized ventro-laterally symmetrical structure made of two asymmetrical bell-shaped cavities (Fig. 35)	*Catharylla chelicerata*
–	Male genitalia with costal arm short, basally wide, tooth shaped, not reaching cucculus tip (Fig. 15). In female genitalia, sterigma forming a pair of shallow pockets opened posterad, antero-ventrad of segment VIII (Fig. 36)	*Catharylla gigantea*
3	Forewing length 7–10 mm, forewing costa slightly ochreous or white on basal 1/2, white on distal 1/2 (Figs 7, 8). In male genitalia, gnathos bent at about 90° angle; uncus less than twice length of tegumen connection, beak shaped (Figs 30, 32)	4
–	Forewing length 10–16 mm, forewing costa with thin ochreous to brown stripe (Figs 1, 4–6). In male genitalia, gnathos more or less straight; uncus more than twice length of tegumen connection (Figs 11, 17, 19, 21)	5
4	Forewing outer margin slightly produced at apex, median transverse line without spots at 1/3 and 2/3 (Fig. 7). In male genitalia, costal arm of valva simple, narrow; sacullus heavily sclerotized, large, laterally with string of sclerotized spines with 2–3 longer basal spines pointing downward (Fig. 30). In female genitalia, sterigma forming mustachio-shaped double rounded cavities set with short spines (Fig. 40)	*Catharylla mayrabonillae*
–	Forewing outer margin not produced at apex, median transverse line with two clearly pronounced spots at 1/3 and 2/3 (Fig. 8). In male genitalia, costal arm of valva with two well-separated projections; sacullus not strongly sclerotized, densely setose, apically rounded (Fig. 32). In female genitalia, sterigma forming double rounded pockets opened posterad (Fig. 41)	*Catharylla paulella*
5	Hindwing white (Fig. 1). In male genitalia, costal arm of valva double, with ventral arm tubular, apically pointing upward, and dorsal arm slightly shorter, flattened, apically rounded; transtilla absent; vesica with one cornutus (Fig. 11). In female genitalia, sternite VIII ventrally connecting, with latero-ventral projections (Fig. 34)	*Catharylla bijuga*
–	Hindwing cream colored or yellowish (Figs 4–6). In male genitalia, costal arm of valva simple; transtilla present; vesica without cornutus, or with crest of cornuti (Figs 17–22). In female genitalia, sternite VIII narrowing ventrally, ventrally not connected, without latero-ventral projection (Figs 37–39)	6
6	Forewing median transverse line more or less straight, shortly curved inward in costal 1/3 (Fig. 4). In male genitalia, transtilla laterally with short, narrow sclerotized arms with pointed tips, projecting posterad, and medially with pair of brushes directed medio-ventrally (Fig. 17). In female genitalia, anterior angle of sternite VIII projected downward into more or less rounded projection covered with short spinules of same length (Fig. 37)	*Catharylla tenellus*
–	Forewing median transverse line not regular, slightly curved outward at M1 and CuA2, curved inward in costal 1/3 (Figs 5–6). In male genitalia, transtilla forming pair of sclerotized arms slightly bent inward with longitudinal row of spines ventrally or medially (Figs 19, 21). In female genitalia, anterior angle of sternite VIII not projected or projected anterad (Figs 38, 39)	7
7	Forewing median transverse line without short marked triangular dent at CuA2 (Fig. 5). In male genitalia, uncus bifid and grooved in distal 1/5; transtilla forming pair of sclerotized arms slightly bent inward distally, ventrally with row of short spines increasing in size from base to apex; juxta regularly narrowing toward apex; vesica with row of 6–7 cornuti (Figs 19, 20). In female genitalia, anterior angle of sternite VIII not projected; anterior apophyses quadrangular, anvil shaped (Fig. 38)	*Catharylla coronata*
–	Forewing median transverse line with short triangular dent at CuA2 (Fig. 6). In male genitalia, uncus apically indented medially; transtilla forming pair of sclerotized arms strongly bent inward in distal 1/4 and with string of long spines of same length medially along it; juxta strongly narrowing in distal 1/4; vesica without cornuti (Figs 21, 22). In female genitalia, anterior angle of sternite VIII projected anterad into rounded projection covered with short spinules; anterior apophyses spine-like (Fig. 39)	*Catharylla serrabonita*

### COI barcode sequences and genetic distances within *Catharylla*

The number of bases obtained for each barcode sequence is given in Table 3 and the genetic distances between specimens in Table 5.

As most of the data were restricted to few sequence samples per species, and some of the sequences came from the same populations, we don’t have the definitive picture of the intraspecific variation in the COI barcode sequences of *Catharylla* species. We observe a relatively high divergence between different barcode haplotypes in *Catharylla bijuga* (5.05%), *Catharylla mayrabonillae* (4.34%), *Catharylla serrabonita* (3.24%) and *Catharylla tenellus* (3.34%), sometimes possibly associated with differences in morphological characters (see Notes of the descriptions of *Catharylla bijuga*, *Catharylla tenellus*) and with geographical distances. The divergence between *Catharylla chelicerata* samples LEP 1703 and LEP 1704 is of 0.15% (1 base) because they are issued from the same population. Sample LEP 1290 differs from samples LEP 1703 and LEP 1704 respectively by 0.62 and 0.46%. We observe no variation between samples LEP 1708, LEP 1709, LEP 1710 and LEP 1888 because they are all issued from the same population. The inter-specific variation in COI barcode sequences (6.29–16.84%) is always higher than the intraspecific variation (0.15–5.05%).

### Morphological results

The 21 analysed characters are listed in Table 6.

### Phylogenetic results
Phylogenetic analysis of *Catharylla* and related genera based on molecular data

The monophyly of *Catharylla* is supported by the analysis of mol_1 and the combined Bayesian analysis (BS support = 90, PP = 0.95) and by the analyses of nucl_1 and nucl_2 (*Catharylla chelicerata*, *Catharylla mayrabonillae*, *Catharylla serrabonita* and *Catharylla tenellus* represented). Three synapomorphies (11:1, 17:1, 19:1, with one reversal to the ancestral state in the *chelicerata* group for character 11) and one non-unique apomorphy (16:1, apomorphy observed in the two outgroups as well) support the group. The *mayrabonillae* group is well supported in all analyses (BS supports = 100 in mol_1 and mol_2 analyses, PP = 1.00 in the combined Bayesian analysis) except in the morphology-based analysis (BS support = 60), because no clear synapomorphy was found for the group. However, two non-unique synapomorphies and one reversal (observed only once in *Catharylla*) are observed (9:1, 12:1, 19:0). The *tenellus* group is well supported by the morphology-based analysis (BS support = 97) with four clear synapomorphies (5:1, 7:3, 10:1, 20:1) and two reversals (3:0, 15:0), but show no support in other analyses, probably because only the barcode sequence was available for *Catharylla coronata*. The closer relationship between *Catharylla serrabonita* and *Catharylla tenellus* within the *tenellus* group is supported by the combined Bayesian analysis and by one non-unique apomorphy (but unique in *Catharylla*). The *tenellus* group as represented in the analyses of nucl_1 and nucl_2 (*Catharylla serrabonita* + *Catharylla tenellus*) is well supported (BS supports of 99 and 93, respectively). The *chelicerata* group is well supported by the morphology-based analysis (BS support = 96) and the combined Bayesian analysis (PP = 1.00). The group shows two synapomorphies (8:1, 9:2), one non-unique apomorphy (7:1) and one reversal (11:0). Unfortunately, no sequence was available for *Catharylla gigantea*, thus we cannot compare the morphology with the molecular-based analyses. The closer relationships between the *chelicerata* and the *mayrabonillae* groups is strongly supported by the nucl_1 analysis (BS support = 100) and the combined Bayesian analysis (PP = 1.00). Two reversals (4:0, 6:0) support the group. The position of *Catharylla bijuga* as sister group of the other *Catharylla* species is weakly supported by the combined Bayesian analysis (PP = 0.90), and show no support in other analyses. The position of *Micrelephas pictellus* as sister group of *Catharylla* is supported by the analysis of mol_2 and nucl_2 (respective BS supports of 91 and 100). This node is supported by one synapomorphy (6:1, with one reversal in the *chelicerata* + *mayrabonillae* clade). Node 10 (*Argyria lacteella*+ *Micrelephas pictellus*+ *Catharylla*) is supported by the analysis of mol_1 (BS support = 100) and the combined Bayesian analysis (PP = 1.00). One synapomorphy (2:1) supports the group. The monophyly of the two *Crambus* species chosen as outgroups is well supported in the mol_1 analysis (BS support of 100). The settings of MrBayes do not allow to choose two outgroups, therefore the monophyly of the two *Crambus* species is not supported by the combined Bayesian analysis. Three non-unique synapomorphies support the genus based on these two species (7:1, 12:1, 16:1). The synapomorphies of the different groups highlighted by the phylogeny are reported here below:

*Chelicerata* group (node 3)

8:1. Apex of valva quadriangular, truncated

9:2. Gnathos regularly curved

*Tenellus* group (node 5)

5:1. Presence of a dorsal furrow on the uncus

7:3. Apex of uncus slightly bifid

10:1. Transtilla present

20:1. Postvaginal sterigma absent

*Catharylla* (node 8)

11:1. Lateroventral projections on juxta

17:1. Posterior apophyses/papillae anales < 0.5

19:1. Ventral membrane of segment VIII with tiny setae

*Catharylla* + *Micrelephas* (node 9)

6:1. Uncus dorsally bare, or with few setae

*Catharylla* + *Micrelephas* + *Argyria* (node 10)

2:1. Length of labial palpi/eye diameter < 2/1

### Geographical distribution and biogeography

*Catharylla* occurs northward from Middle America with locality records in Costa Rica (Area de Conservacion Guanacaste) at a latitude of 10°54 N, southward to Rio Vermelho (Brazil, Santa Catarina) at a latitude of 27°30 S, from sea level up to 1300 m (Brazil, Minas Gerais, Serra do Caraça). Within *Catharylla*, species and species groups show distinct distribution patterns. The *chelicerata* group is widespread in the Northern Amazonian rainforest of Brazil, and in the three Guyanas (Fig. 43). The *tenellus* group is restricted to the south-eastern Atlantic coast of Brazil in the Atlantic Forest, which is formed by tropical moist forests (Thomas and Britton 2008) (Fig. 45). These two groups seem to be restricted to moist forest habitats. The sister species *Catharylla mayrabonillae* and *Catharylla paulella* show an allopatric distribution: *Catharylla mayrabonillae* is widespread northwards up to Costa Rica and southwards down to Brasilia (15°47 S), whereas *Catharylla paulella* is widespread from Feira Nova (07°00 S) down to São Paulo (23°35 S) (Fig. 44). They are both recorded from the Federal District, and from Maranhão (Feira Nova). Both species show wide geographical distribution covering a large range of ecosystems: *Catharylla mayrabonillae* occurs in the tropical moist forests of the Carribean, the Northwestern South American and the Amazonian subregions, as well as in the Cerrado province of Central Brazil (records from Brasilia, Distrito Federal) (Figs 43, 44), where savannahs predominate with a semi-humid tropical climate and a pronounced dry period during winter (april-september) (Ratter et al. 1997). *Catharylla paulella* occurs in the Cerrado (Planaltina, Distrito Federal), in the Chaco formation (Urucum, Matto Grosso, Brazil and Provincia del Sara, Bolivia) (Figs 43, 44), which is a hot and dry area with xerothermic deciduous forests (Candollea 1993). *Catharylla bijuga* is restricted to the Guyana province. The distribution areas of the *chelicerata* group and that of *Catharylla bijuga* are allopatric with respect to that of the *tenellus* group. The dry diagonal (Prado and Gibbs 1993) formed by the Caatinga, the Cerrado and the Gran Chaco, highlighted by the low rainfalls rates (represented in yellow, orange and brown on Fig. 43), constitutes the climatic barrier between the Atlantic and the Amazonian Forests. Both barcode haplotypes of *Catharylla bijuga* are from the Amazonian Forest, but they are distant from each other by about 1290 km and from different provinces: haplotype BC MTD 1839 is from the Varzea province, near the Rio Negro (Brazil, Parque nacional do Jaú) while haplotype BC MTD 1840 is from the Humid Guyana province (French Guyana, Roura). *Catharylla mayrabonillae* barcode haplotype 07-SRNP-113921 is found in the Carribean subregion, whereas haplotype LEP 1126 is found in the Amazonian subregion. The barcode haplotypes of the *Catharylla serrabonita* populations from the two coastal localities of Linhares and Porto Seguro are more closely related, whereas the haplotype from the population of the forested hills of the Serra Bonita Reserve clearly diverges from the other two (Fig. 46).

## Discussion

### Reliability of phylogenetic analyses

The analyses of nucl_1 and nucl_2 datasets brought better BS supports than the analyses of mol_1 and mol_2 on nodes 1, 6, 9, and for the monophyly of *Catharylla* (nodes 7 & 8). The reduced number of taxa (7 taxa in nucl datasets vs 11 in mol datasets, with 4 *Catharylla* species in nucl_1 and nucl_2 vs 7 in mol_1 and mol_2), as well as the quality of the datasets (complete sets of genes for nucl_1 and nucl_2 vs partly complete sets of genes in mol_1 and mol_2) explain the better results obtained in nucl_1 and nucl_2, because taxa with incomplete data tend to lower the resolution and the bootstrap supports of the tree (Regier et al. 2009). The barcode sequence evolves rapidly and therefore fails to accurately reconstruct phylogenetic relationships. The analysis of a higher number of genes would increase the support values (Wahlberg and Wheat 2008). The morphology-based analysis yielded good support for two species groups within *Catharylla*, but failed to reconstruct the phylogenetic relationships between different *Catharylla* species groups and between *Catharylla* and other genera. The weak number of characters of the matrix used (21, reported in Table 6) compared to the number of taxa, as well as the homoplastic characters explain the lack of bootstrap-supported groups (Bremer et al. 1999). The combined Bayesian analysis yielded good supports within *Catharylla* and between *Catharylla* and other genera.

### Taxa positions on the phylogenetic tree

Our molecular-based analyses failed to place with certainty two of the four taxa lacking nuclear sequences (*Catharylla bijuga* and *Catharylla coronata*) because of the great divergence of these sequences from those of other *Catharylla* species. However, the clear support brought by the morphological analysis places *Catharylla coronata* together with *Catharylla serrabonita* and *Catharylla tenellus*. The neighbor-joining (NJ) analysis of barcode sequences of *Catharylla* species (not represented here) places *Catharylla coronata* together with *Catharylla serrabonita* (divergence of 7.59% with the sequence BC MTD 1843) and *Catharylla tenellus* (divergence of 7.3% with the sequence BC MTD 1842), and thus corroborates the findings of the morphology-based analysis. Moreover, the species is morphologically very similar to *Catharylla serrabonita* especially regarding the transtilla in the male genitalia. The basal position of *Catharylla bijuga* in *Catharylla*, as sister taxon of other *Catharylla* species, is doubtful (PP = 0.90) and may be an artefact due to the great divergence of the barcode sequence (the lowest divergence with other *Catharylla* species is of 9.7% with sample BC MTD 1843 of *Catharylla serrabonita*). The supports brought by the combined Bayesian analysis have to be carefully considered since the results are sensitive to small variations of the taxon and character sampling, and the posterior probabilities tend to overestimate the strongness of the nodes (Buckley et al. 2002, Whittingham et al. 2002). For *Catharylla bijuga* and *Catharylla coronata*, a complete gene dataset would be needed because the barcode sequence alone is not sufficient to undoubtfully assess their position within *Catharylla*. The closer relationships of the *chelicerata* and the *mayrabonillae* groups (Fig. 42, node 6) is well supported by the analysis of nucl_1 and by the combined Bayesian analysis (BS support = 100, PP = 1.00), but a decrease of the BS support is observed when comparing mol_1 to mol_2, and nucl_1 to nucl_2, showing that the third codon position of the COI gene supports this node. This suggests a rapid evolution of this group where mutations accumulated only at the less constrained third codon position, or this could result from convergent substitutions in each lineage. *Micrelephas pictellus* is here placed as sister group of *Catharylla*, an unexpected topology, given that *Catharylla* has been placed in the Argyriini (type genus: *Argyria*) along with *Argyria*, *Urola* and *Vaxi*, and *Micrelephas* in Crambini (Landry 2003). The Argyriini as defined here are not monophyletic and would need to be redescribed, whether to include *Micrelephas*, or to exclude *Catharylla*. A more extensive analysis of the tribe with *Urola*, *Vaxi* and more genera of Crambini would be desirable to assess the validity and the composition of the Argyriini. The position of *Micrelephas* as sister group of *Catharylla* is subject to caution as only three Crambinae genera of the 179 currently recognized (Nuss et al. 2013) are represented in the analyses. The short labial palpi (i. e. the length of labial palpi/eye diameter < 2/1), which constitutes a synapomorphy for node 10 is observed in few other genera of Crambinae (*Euchromius*, *Myelobia* and *Urola*, see Landry 1995), for which long, porrect palpi are the norm.

### Species delimitation and possible cryptic species in *Catharylla*

The intra-specific divergence in barcode sequences within *Catharylla* (Table 5; distances mapped on Figs 44, 45 and 46) is higher than the threshold of 2–3% predicted to delimit the species level in Lepidoptera according to Hebert et al. (2003), with intraspecific divergence often representing overlooked species (Hebert et al. 2003, Mutanen et al. 2012). The maximum intraspecific COI barcode divergences observed in *Catharylla bijuga* (5.05%), *Catharylla mayrabonillae* (4.34%), *Catharylla serrabonita* (3.24%) and *Catharylla tenellus* (3.34%) could therefore suggest that these different barcode haplotypes represent different species. Some morphological variation in male genitalia was found in *Catharylla serrabonita* and *Catharylla tenellus* (Figs 23–29) haplotypes, but none in *Catharylla bijuga* and *Catharylla mayrabonillae*, which is counter-intuitive given that the latter two species have more divergence than the other two. Moreover, none of the intraspecific divergence observed in *Catharylla* is higher than the interspecific divergence. A greater number of specimens would be required to properly investigate the COI and nuclear gene variation along with morphological and ecological variations to judge if these COI barcode haplotypes and morphological variations represent meaningful differences linked with speciation or incipient speciation or if these species are really older than average. For now, given the available data, we are confident in our decision to recognize the eight species treated here in genus *Catharylla*.

## Biogeography

The different species groups of *Catharylla* highlighted by the phylogenetic analyses show distinct distribution patterns, with species widely spread, such as *Catharylla mayrabonillae* and *Catharylla paulella* (Fig. 44) and other species confined to smaller areas, such as *Catharylla bijuga* (Fig. 45), and the *chelicerata* and *tenellus* species groups (Figs 43 & 45). The humid formations of the Amazonian and Atlantic Forests constituted two different refuges separated by the Caatinga and the Cerrado xerothermic formations, and vicariant evolutionary processes in plants (Rizzini 1997; Perret et al. 2012) and in birds (Eberhard and Bermingham 2005) have been documented. The distribution of *Catharylla mayrabonillae* and *Catharylla paulella* (Fig. 44), respectively northwards and southwards of the dry diagonal could also result from the split of a former population between the Amazon and the Atlantic Forests where speciation events occurred during dry spells, with expansion of the species afterwards. The patterns of distribution of the *chelicerata* group (Fig. 43) suggest that its diversification might have occured in the Amazonian Forest. The two species are sympatric as they are both recorded from Reserva Ducke. Similarly, *Catharylla coronata* and *Catharylla tenellus* are sympatric (both collected in Rio de Janeiro and São Paulo), as well as *Catharylla serrabonita* and *Catharylla tenellus* (both collected in Porto Seguro), while *Catharylla coronata* and *Catharylla serrabonita* are vicariant, *Catharylla serrabonita* being distributed north of Linhares, whereas *Catharylla coronata* is found south of this locality. The patterns of distribution suggest that the diversification of the *tenellus* group might have occurred in the Atlantic Forest, or have occurred in a wider area that reduced afterwards. In *Catharylla tenellus*, two forms of male valva linked to different COI barcode sequences seem to be geographically separated, with the form from Ubatuba (associated to barcode sequence BC MTD 1842), Bertioga, São Paulo (São Paulo State), and Caraça (Minas Gerais) occurring more to the south than the form from Porto Seguro (associated with barcode sequences BC MTD 1709, 1710, 1711, 1888). The third form, observed in Caraça (Minas Gerais) suggests that this locality could represent a point of contact between the two other forms. Regarding *Catharylla serrabonita*, the locality of Serra Bonita is a moist forest of middle elevation (800 m) (Fig. 46), while the localities of Porto Seguro and Linhares are part of a drier biome along the Atlantic Coast (V. O. Becker, pers. comm.). Therefore, the genetic distance found between these populations of this species could barely be explained by the distance between the two localities (130 km between Serra Bonita and Porto Seguro), but could be related to different ecological conditions.

## Supplementary Material

XML Treatment for Catharylla

XML Treatment for Catharylla bijuga

XML Treatment for Catharylla chelicerata

XML Treatment for Catharylla gigantea

XML Treatment for Catharylla tenellus

XML Treatment for Catharylla coronata

XML Treatment for Catharylla serrabonita

XML Treatment for Catharylla mayrabonillae

XML Treatment for Catharylla paulella

## Appendix I

**Table 1.** Coordinates of the localities pertaining to the *Catharylla* specimens studied.

Country	Locality	Province / State / Subdivision	latitude, longitude	
Bolivia	?	Sara	-16°55, -63°37	
Brazil	Belem	Pará	-1°27, -48°30	
	Bertioga	São Paulo	-23°51, -46°8	
	Cacaulândia	Rondônia	-10°20, -62°53	
	Capitão Poço	Pará	-1°44, -47°3	
	Caraça	Minas Gerais	-20°07, -43°28	
	Castro	Paraná	-24°47, -50°0	
	Corcovado	Rio de Janeiro	-22°57, -43°13	
	Curitiba	Paraná	-25°25, -49°16	
	Fazenda Rancho Grande	Rondônia	-10°18, -62°52	
	Feira Nova	Maranhão	-07°00, -46°26	
	Fonte Boa	Amazonas	-2°31, -66°05	
	Ibateguara	Alagoas	-8°58, -35°55	
	Lago Teffé	Amazonas	-3°27, -64°53	
	Linhares	Espírito Santo	-19°23, -40°3	
	Manaus	Amazonas	-3°7, -60°0	
	Mirapinima	Amazonas	-2°10, -61°9	
	Nova Friburgo	Rio de Janeiro	-22°17, -42°32	
	Nova Olinda do Norte	Amazonas	-3°54, -59°05	
	Parque Nacional de Jaú	Amazonas	-1°57, -61°49	
	Planaltina	Federal District	-15°36, -47°40	
	Porto Seguro	Bahia	-16°27, -39°3	
	Quatro Barras	Paraná	-25°22, -49°4	
	Reserva Ducke	Amazonas	-2°55, -59°58	
	Rio Branco	Acre	-9°58, -67°48	
	Rio Negro	Paranà	-26°6, -49°48	
	Rio Vermelho	Santa Catarina	-27°29, -48°24	
	Sao Luiz do Paraitinga	São Paulo	-23°13, -45°18	
	São Paulo	São Paulo	-23°32, -46°38	
	Serra Bonita	Bahia	-15°23, -39°33	
	Ubatuba	São Paulo	-23°26, -45°5	
	Urucum	Mato Grosso do Sul	-19°8, -57°38	
Colombia	Buenaventura	Valle del Cauca	3°49, -77°00	
Costa Rica	Estacion Caribe	Alajuela	10°54'06, -85°16'30	
	Pitilla	Guanacaste	10°59'21, -85°25'32	
	Parque Nacional Arenal	Alajuela	10°26, -84°44	
Ecuador	Misahualli	Napo	-1°1, -77°40	
	Santa Clara	Pastaza	-1°15, -77°53	
French Guiana	Beauséjour		4°42, -52°23	
	Cayenne		4°55, -52°19	
	Montagne des Chevaux		4°43, -52°25	
	Nancibo		4°40, -52°28	
	Oyapock, Pied Saut		3°48, -51°53	
	Parcelles CIRAD de Combi		5°18, -52°55	
	Roura, road to Crique Gabrielle		4°44, -52°19	
	Roura, Camp Patawa		4°33, -52°9	
	Route d’Apatou pk 25.5		5°15, -54°13	
	Route de Saut Léodate		4°55, -52°33	
	Saint-Jean-du-Maroni		5°24, -54°4	
	Saint-Laurent-du-Maroni		5°30, -54°01	
Guyana	Malali	Upper Demerara–Berbice	5°37, -58°21	
	New River	East Berbice–Corentyne	3°21, -57°35	
	Omai	Cuyuni–Mazaruni	5°25, -58°45	
	Potaro	Potaro–Siparuni	5°22, -59°7	
Panama	Rio Trinidad	Nuevo Emperador	8°43, -79°58	
Peru	Aguaytía	Ucayali	-9°20, -75°30	
	Panguana	Huánuco	-9°36, -74°56	
	Yurimaguas	Loreto	-5°54, -76°7	
Suriname	Gelderland	Para	5°27, -54°59	
	Kabo	Sipaliwini	5°16, -55°44	
	Kabo Kreek	Sipaliwini	5°8, -57°16	

## Appendix II

Molecular material and techniques.

**Table 1.** PCR mix for respective genes and primer pairs.

Product	LCO/Pat, LCO/Nancy, Jerry/ Pat (COI) HybLepWg1 / HybLepWg2 (wingless)	LCO/K699, Ron/Nancy, Jerry/Mila, Brian/Pat (COI), HybLepWg1/ HybLepWg2 (wingless), Oscar-6143 /Bosie-6144 (EF-1α)	HybLepWg1/HybLepWg2 (wingless), Oscar-6143/ Bosie-6144 (EF-1α)
H2O (ultraclean)	15.9 μl	12.55 μl	11.95 μl
10X Taq buffer (15 mM MgCl_2_)	2.5 μl	2.5 μl	2.5 μl (10X OptiBuffer)
MgCl_2_ (25 mM)	1 μl	0.75 μl	0.75 μl (50 mM)
Forward primer (10 pmol/μl)	0.5 μl	1.2 μl	1.5 μl
Reverse primer (10 pmol/μl)	0.5 μl	1.2 μl	1.5 μl
dNTP (each 10 mM)	0.5 μl	2.5 μl	2.5 μl
Taq DNA polymerase (5 units/μl)	0.1 μl	0.3 μl	0.3 μl (BIO-X-ACT Short TaqDNA polymerase; 4 units/μl)
DNA	4 μl	4 μl	4 μl

**Table 2.** PCR programmes for COI, wingless and EF-1α.

Gene		COI, wingless	wingless	EF-1α
Polymerase		Taq polymerase	BIO-X-ACT Short Taq	Taq polymerase
programme	initial:	95 °C (5 min)	95 °C (5 min), 48 °C (1 min)	95 °C (5 min)
	40 cycles:	94 °C (30 sec)	95 °C (30 sec)	94 °C (30 sec)
		48 °C (30 sec)	51 °C (30 sec)	51 °C (30 sec)
		72 °C (1 min 30)	70 °C (1 min)	72 °C (1 min 30)
	final:	72 °C (10 min)	70 °C (10 min)	72 °C (10 min)
	cooling:	8 °C (∞)	8 °C (∞)	8 °C (∞)

**Table 3.** PCR-mix for sequencing reaction.

Product	low DNA concentration	high DNA concentration
cleaned PCR-product	4 μl	1 μl
H_2_O (ultraclean)	2 μl	5 μl
Forward primer (10 pmol/μl)	1 μl	1 μl
T-mix (2.5×)	1 μl	1 μl
Buffer (5×)	2 μl	2 μl

## References

[B1] Bleszynski S (1962) Studies on the Crambidae (Lepidoptera). Part XXXVII. Changes in the nomenclatory [sic] of some Crambidae with the descriptions of new genera and species. Polskie Pismo Entomologiczne 32(1): 5–48, pl. 7.

[B2] Bleszynski S (1967) Studies on the Crambinae (Lepidoptera). Part 44. New Neotropical genera and species. Preliminary check-list of Neotropical Crambinae. Acta Zoologica Cracoviensia 12(5): 39–110, pls. 11–14.

[B3] Bleszynski S Collins RJ (1962) A short catalogue of the world species of the Family Crambidae (Lepidoptera). Acta Zoologica Cracoviensia 7: 197-389.

[B4] Börner C (1925) Lepidoptera, Schmetterlinge. In: Brohmer P (Ed) Fauna von Deutschland. Quelle & Meyer, Berlin, 561 pp.

[B5] Bremer B Jansen RK Oxelman B Backland M Lantz H Kim KJ (1999) More characters or more taxa for a robust phylogeny – Case study from the coffee family (Rubiaceae). Systematic Biology 48: 413-435. doi: 10.1080/106351599260085 12066290

[B6] Buckley TR Arensburger P Simon C Chambers GK (2002) Combined data, Bayesian phylogenetics, and the origin of the New Zealand cicada genera. Syst. Biol. 51: 4-18. doi: 10.1080/106351502753475844 11943089

[B7] Candollea V (1993) What is Gran Chaco vegetation in South America? A review. Contribution to the study of flora and vegetation of Chaco 48: 145-172.

[B8] Dyar HG (1914) Report on the Lepidoptera of the Smithsonian Biological Survey of the Panama Canal Zone. Proceedings of the United States National Museum, Washington 47(2050): 139-350.

[B9] Eberhard JR Bermingham E (2005) Phylogeny and comparative biogeography of Pionopsitta parrots and Pteroglossus toucans. Molecular Phylogenetics and Evolution 36(2): 288-304. doi: 10.1073/pnas.0406166101 15955511

[B10] Hajibabaei M Janzen DH Burns JM Hallwachs W Hebert PD (2006) DNA barcodes distinguish species of tropical Lepidoptera. Proceedings of the National Academy of Sciences USA 103: 968-971. doi: 10.1073/pnas.0510466103 PMC1327734 16418261

[B11] Hall TA (1999) BioEdit: a user–friendly biological sequence alignment editor and analysis program for Windows 95/98/NT. Nucl. Acids. Symp. Ser. 41: 95-98.

[B12] Hampson GF (1896) On the classification of the Schoenobiinae and Crambinae, two subfamilies of moths, of the family Pyralidae. Proceedings of the General Meetings for Scientific Business of the Zoological Society of London 1895(4): 897-974.

[B13] Hasenfuß I (1960) Die Larvalsystematik der Zünsler. Academie Verlag, Berlin.

[B14] Hebert PDN Cywinska A Ball SL Dewaard JR (2003) Biological identifications through DNA barcodes. Philosophical Transactions of the Royal Society of London, Series B, Biological Sciences 270: 313-321. doi: 10.1098/rspb.2002.2218 PMC1691236 12614582

[B15] Hebert PDN Penton EH Burns JM Janzen DH Hallwachs W (2004) Ten species in one: DNA barcoding reveals cryptic species in the neotropical skipper butterfly Astraptes fulgerator. Proceedings of the National Academy of Sciences of the United States of America. doi: 10.1073/pnas.0406166101 PMC522015 15465915

[B16] Hijmans R Guarino J Jarvis A O´Brien R Mathur P (2011) DIVAGIS 7.4.0.1.

[B17] Hübner J (1808–1818) Zuträge zur Sammlung exotischer Schmettlinge [sic], bestehend in Bekundigung einzelner Fliegmuster neuer oder rarer nichteuropäischer Gattungen. Augsburg, [1]–[3]–4–6–[7]–8–32–[33]–[40], pl. [1]–[35].

[B18] Huelsenbeck JP Ronquist F (2001) MRBAYES: Bayesian inference of phylogenetic trees. Bioinformatics 17: 754-755. doi: 10.1093/bioinformatics/17.8.754 11524383

[B19] Hundsdörfer AK Rubinoff D Attié M Wink M Kitching IJ (2009) A revised molecular phylogeny of the globally distributed hawkmoth genus Hyles (Lepidoptera: Sphingidae), based on mitochondrial and nuclear DNA sequences. Molecular Phylogenetics and Evolution 52: 852-865. doi: 10.1016/j.ympev.2009.05.023 19482093

[B20] Ivanova NV deWaard JR Hebert PDN (2006) An inexpensive, automation-friendly protocol for recovering high-quality DNA. Molecular Ecology Notes 6: 998–1002. doi: 10.1111/j.1471-8286.2006.01428.x

[B21] Knölke S Erlacher S Hausmann A Miller MA Segerer AH (2005) A procedure for combined genitalia extraction and DNA extraction in Lepidoptera. Insect Systematics and Evolution 35: 401-409. doi: 10.1163/187631204788912463

[B22] Kristensen NP (2003) Lepidoptera, Moths and Butterflies, 1 Evolution, Systematics, and Biogeography. Handbuch der Zoologie 4(35). Walter de Gruyter, Berlin, New York.

[B23] Landry B (1993) The types of North American Crambinae (Lepidoptera: Pyralidae) in the Natural History Museum, London. The Canadian Entomologist 125: 1077-1090. doi: 10.4039/Ent1251077-6

[B24] Landry B (1995) A phylogenetic analysis of the major lineages of the Crambinae and of the genera of Crambini of North America (Lepidoptera: Pyralidae).

[B25] Landry B (2003) Revision of the Neotropical genus Micrelephas (Lepidoptera: Pyralidae: Crambinae). Tropical Lepidoptera, Gainesville 11: 13-27.

[B26] Latreille PA (1810) Considérations générales sur l’Ordre naturel des Animaux composant les Classes des Crustacés, des Arachnides et des Insectes; avec un Tableau méthodique de leurs Genres, disposés en Familles. F. Schoell, Paris, 444 pp.

[B27] Miller MA Pfeiffer W Schwartz T (2010) Creating the CIPRES Science Gateway for inference of large phylogenetic trees. Proceedings of the Gateway Computing Environments Workshop (GCE), New Orleans, LA, 1–8. doi: 10.1109/GCE.2010.5676129

[B28] Minet J (1982) Les Pyraloidea et leurs principales divisions systématiques. Bulletin de la Société Entomologique de France 86: 262-280.

[B29] Morrone JJ (2006) Biogeographic areas and transition zones of Latin America and the Caribbean islands based on panbiogeographic and cladistic analyses of the entomofauna. Annual Review of Entomology 51: 467-94. doi: 10.1146/annurev.ento.50.071803.130447 16332220

[B30] Müller J Müller KF Neinhuis C Quandt D (2011) PhyDE – Phylogenetic Data Editor. http://www.phyde.de

[B31] Munroe EG (1995) Crambidae (Crambinae, Schoenobiinae, Cybalomiinae, Linostinae, Glaphyriinae, Dichogaminae, Scopariinae, Musotiminae, Midilinae, Nymphulinae, Odontiinae, Evergestinae, Pyraustinae). In: Heppner JB (1995) Atlas of Neotropical Lepidoptera. Checklist: Part 2. Hyblaeoidea-Pyraloidea-Tortricoidea 3. Association for Tropical Lepidoptera & Scientific Publishers, Gainesville, 34–79.

[B32] Munroe EG Solis MA (1998) The Pyraloidea. In: Kristensen NP (Ed) Lepidoptera, Moths and Butterflies, Volume 1: Evolution, systematics, and biogeography. Handbook of Zoology. Volume IV Arthropoda: Insecta, Part 35. Walter de Gruyter, Berlin & New York, 233-256.

[B33] Mutanen M Wahlberg K Kaila L (2010) Comprehensive gene and taxon coverage elucidates radiation patterns in moths and butterflies. Proceedings of the Royal Society, B, 277: 2839–2849. doi: 10.1098/rspb.2010.0392 PMC2981981 20444718

[B34] Mutanen M Hausmann A Hebert PDN Landry JF de Waard JR Huemer P (2012) Allopatry as a Gordian Knot for Taxonomists: Patterns of DNA Barcode Divergence in Arctic-Alpine Lepidoptera. PLoS ONE 7(10): e47214. doi: 10.1371/journal.pone.0047214 23071761 PMC3469483

[B35] Nuss M Landry B Vegliante F Tränkner A Mally R Hayden J Segerer A Li H Schouten R Solis MA Trofimova T De Prins J Speidel W (2003–2013) Global Information System on Pyraloidea. http://www.pyraloidea.org

[B36] Nylander JAA (2004) MrAIC.pl. Program distributed by the author Evolutionary Biology Centre, Uppsala University.

[B37] Perret M Chautems A De Araujo AO Salamin N (2012) Temporal and spatial origin of Gesneriaceae in the New World inferred from plastid DNA sequences. Botanical Journal of the Linnean Society. doi: 10.1111/j.1095-8339.2012.01303.x

[B38] Prado DE Gibbs PE (1993) Patterns of species distributions in the dry seasonal forests of South America. Annals of the Missouri Botanical Garden 80(4): 902–927.

[B39] Ratter JA Ribeiro JF Bridgewater S (1997) The Brazilian Cerrado vegetation and threats to its biodiversity. Annals of Botany 80: 223-230. doi: 10.1006/anbo.1997.0469

[B40] Regier JC Zwick A Cummings MP Kawahara AY Cho S Weller S Roe A Baixeras J Brown JW Parr C Davis DR Epstein M Hallwachs W Hausmann A Janzen DH Kitching IJ Solis MA Yen SH Bazinet AL Mitter C (2009) Toward reconstructing the evolution of advanced moths and butterflies (Lepidoptera: Ditrysia): an initial molecular study. BioMed Central Evolutionary Biology 9: 280-301. doi: 10.1186/1471-2148-9-280 19954545 PMC2796670

[B41] Regier JC Mitter C Solis MA Hayden JE Landry B Nuss M Simonsen TJ Yen SH Zwick A Cummings MP (2012) A molecular phylogeny for the pyraloid moths (Lepidoptera: Pyraloidea) and ist implications for higher-level classification. Systematic Entomology 37: 635-656. doi: 10.1111/j.1365-3113.2012.00641.x

[B42] Rizzini CT (1997) Tratado de fitogeografia do Brasil. Âmbito Cultural Edições Ltda, Rio de Janeiro, 2ª Ed., 747 pp.

[B43] Schaus W (1922) New species of Pyralidae of the subfamily Crambinae from tropical America. Proceedings of the Entomological Society of Washington 24: 127-145.

[B44] Stamatakis A (2006) RAxML-VI-HPC: maximum likelihood-based phylogenetic analyses with thousands of taxa and mixed models. Bioinf 22: 2688-2690. doi: 10.1093/bioinformatics/btl446 16928733

[B45] Stamatakis A Hoover P Rougemont J (2008) A rapid Bootstrap Algorithm for the RaxML Web-Servers. Systematic Biology 75(5): 758–771. doi: 10.1080/10635150802429642 18853362

[B46] Swofford DL (2002) PAUP*. Phylogenetic Analysis Using Parsimony (*and Other Methods). Version 4. Sinauer Associates, Sunderland, Massachusetts.

[B47] Thomas WW Britton EG (2008) The Atlantic coastal forest of Northeastern Brazil. The New York Botanical Garden Press, Bronx, N.Y., 586 pp.

[B48] Vanzolini P Heyer WR (1987) Proceedings of a Workshop on Neotropical Distribution Patterns.

[B49] Wahlberg N Wheat CW (2008) Genomic outposts serve the phylogenomic pioneers: designing novel nuclear markers for genomic DNA extractions of Lepidoptera. Systematic Biology 57(2): 231-242. doi: 10.1080/10635150802033006 18398768

[B50] Walker F (1863) Crambites & Tortricites. List of the Specimens of Lepidopterous Insects in the Collection of the British Museum, London 27: 1-286.

[B51] Whittingham LA Slikas B Winkler DW Sheldon FH (2002) Phylogeny of the tree swallow genus, Tachycineta (Aves: Hirundinidae), by Bayesian analysis of mitochondrial DNA sequences. Mol. Phylogenet. Evol. 22: 430-41. doi: 10.1006/mpev.2001.1073 11884168

[B52] Xia X Xie Z (2001) DAMBE: Data Analysis in Molecular Biology and Evolution. Journal of Heredity 92: 371-373. doi: 10.1093/jhered/92.4.371 11535656

[B53] Zeller PC (1839) Versuch einer naturgemäßen Eintheilung der Schaben. Isis von Oken, Leipzig [32] (1–12): 167–219.

[B54] Zeller PC (1863) Chilonidarum et Crambidarum genera et species. Wiegandt & Hempel, Meseritz & Berlin, 1–56.

[B55] Zeller PC (1866) Beschreibung einiger amerikanischen Wickler und Crambiden. Stettiner Entomologische Zeitung 27(4–6): 137–157, 1 pl.

[B56] Zeller PC (1872) Beiträge zur Kenntnis der nordamerikanischen Nachtfalter, besonders der Mikrolepidopteren. Verhandlungen der Zoologisch-Botanischen Gesellschaft in Wien 22: 447–566, pls. 2–3.

[B57] Zeller PC (1877) Exotische Microlepidoptera. Horae Societatis entomologicae Rossicae, St. Petersbourg 13: 1–491, pls 1–6.

[B58] Zeller PC (1881) Columbische Chiloniden, Crambiden und Phycideen. Horae Societatis entomologicae Rossicae, St. Petersbourg 16: 154–256, pls 11–12.

